# Transcriptomic Basis of Serum Resistance and Virulence Related Traits in XDR *P. aeruginosa* Evolved Under Antibiotic Pressure in a Morbidostat Device

**DOI:** 10.3389/fmicb.2020.619542

**Published:** 2021-01-25

**Authors:** Mumina Javed, Benedikt Jentzsch, Maximilian Heinrich, Viola Ueltzhoeffer, Silke Peter, Ulrich Schoppmeier, Angel Angelov, Sandra Schwarz, Matthias Willmann

**Affiliations:** ^1^Interfaculty Institute of Microbiology and Infection Medicine Tübingen, Institute of Medical Microbiology and Hygiene, Tübingen, Germany; ^2^German Center for Infection Research (DZIF), Partner Site Tübingen, Tübingen, Germany; ^3^NGS Competence Center Tübingen (NCCT), Tübingen, Germany; ^4^Eurofins MVZ Medizinisches Labor Gelsenkirchen, Gelsenkirchen, Germany

**Keywords:** multi-drug resistance, biofilm formation, evolutionary trajectories, serum susceptibility, colistin, combination drug therapy, bacterial fitness, automated devices

## Abstract

Colistin is a last resort antibiotic against the critical status pathogen *Pseudomonas aeruginosa*. Virulence and related traits such as biofilm formation and serum resistance after exposure to sub-inhibitory levels of colistin have been underexplored. We cultivated *P. aeruginosa* in a semi-automated morbidostat device with colistin, metronidazole and a combination of the two antibiotics for 21 days, and completed RNA-Seq to uncover the transcriptional changes over time. Strains became resistant to colistin within this time period. Colistin-resistant strains show significantly increased biofilm formation: the cell density in biofilm increases under exposure to colistin, while the addition of metronidazole can remove this effect. After 7 days of colistin exposure, strains develop an ability to grow in serum, suggesting that colistin drives bacterial modifications conferring a protective effect from serum complement factors. Of note, strains exposed to colistin showed a decrease in virulence, when measured using the *Galleria mellonella* infection model. These phenotypic changes were characterized by a series of differential gene expression changes, particularly those related to LPS modifications, spermidine synthesis (*via speH* and *speE*) and the major stress response regulator *rpoS*. Our results suggest a clinically important bacterial evolution under sub-lethal antibiotic concentration leading to potential for significant changes in the clinical course of infection.

## Introduction

*Pseudomonas aeruginosa* is a gram-negative, opportunistic bacterium and a frequent cause of healthcare acquired infections (HAIs). It belongs to the group of ESKAPE pathogens, which consist of six microorganisms, namely *Enterococcus faecium, Staphylococcus aureus*, *Klebsiella pneumoniae, Acinetobacter baumannii*, *Pseudomonas aeruginosa*, and *Enterobacter* spp., with a high tendency for causing challenging, drug-resistant, nosocomial infections (Rice, 2008). *P. aeruginosa* accounts for up to 15.4% of bloodstream infections (BSIs) in intensive care units (ICUs) in Western Europe [Antimicrobial resistance and healthcare-associated infections – Annual epidemiological report 2014 (2012 data)], causing high mortality rates.

One of the drugs of last resort against multi drug resistant (MDR) strains of *P. aeruginosa* is colistin. Colistin has a bactericidal effect: as a cationic cyclic peptide, it is able to bind to anionic lipopolysaccharide (LPS) modules and displace Ca2^+^ and Mg2^+^ from the outer cell membrane of *P. aeruginosa*, leading to disruption in the permeability of the membrane, leaking of cell contents, and cell death (Falagas et al., 2005). Although the spread of colistin resistance is not critical to date, resistance has emerged in some instances worldwide, particularly with the increased reliance on colistin for treating multidrug-resistant gram-negative bacterial infections (Lee et al., 2016). It is crucial that new measures are taken to prevent the development of resistance against this last resort drug, including using microbial evolution experiments to uncover the molecular basis of adaptive evolution. Antibiotics are often given in combination in standard therapeutic regimens, with metronidazole being a common drug partner for the treatment of infections by obligate and facultative anaerobic bacteria. It is effective for the management of intra-abdominal infections, gynecological infections, septicemia, endocarditis, bone, and joint infections, amongst several other types of infections (Löfmark et al., 2010). Metronidazole in treatment inhibits DNA synthesis and DNA damage by oxidation, causing single-strand and double-strand breaks that lead to DNA degradation and cell death. Metronidazole is activated when reduced, with molecules binding non-specifically to bacterial DNA, inactivating the DNA and key enzymes of the pathogen; leading to a high level of DNA breakage (Land and Johnson, 1999). An interesting study found that despite metronidazole having no bactericidal effect on *P. aeruginosa*, *in vitro* exposure to a therapeutic concentration of metronidazole increased the number of mutations through induction of the SOS response, thus leading to emergence of antibiotic resistant bacteria (Hocquet and Bertrand, 2014).

Advances in next-generation sequencing of RNA have enabled the analysis of transcriptional changes that occur in bacteria when continuously grown *in vitro* in presence of sub-lethal doses of antibiotics (McPhee et al., 2003; Hood et al., 2013). These evolutionary studies have been extremely valuable in identifying genes and pathways that confer antibiotic resistance, in cases where this is mediated by chromosomal mutations. However, there is a significant lack of mass or high throughput evolutionary studies. In addition, how the transcriptional changes under antibiotic pressure and combination regimens affect other clinically relevant phenotypes remains poorly explored. Such changes may alter the manifestation and severity of the infection as well as the response to treatment and involve factors such as biofilm formation, immune response evasion through serum resistance and virulence. One study which used single drug treatment and examined populations of *P. aeruginosa* evolved in the presence of sublethal concentrations of ciprofloxacin found significant phenotypic changes such as reduced protease activity and swimming motility, as well as increased levels of quorum-sensing (QS) signal molecules (Wassermann et al., 2016). Their results suggested that evolution in the presence of sublethal concentrations of antibiotics have pleiotropic effects on the phenotypes of pathogens. This may promote persistence of the resistant bacterial populations.

The ability of *P. aeruginosa* to form biofilms is associated with severe infections and significant morbidity and mortality (Mulcahy et al., 2014). Biofilms provide *P. aeruginosa* an enormous advantage by promoting survival on medical devices such as catheters, evasion from the immune system, and tolerance to antimicrobial therapy. Increased biofilm formation has also been found to contribute to increased virulence (Ghadaksaz et al., 2015; Maurice et al., 2018). Other virulence factors of *P. aeruginosa* include, amongst others, toxins, exoproteases, phospholipases, the presence of type IV pili and flagella (Ballok and O’Toole, 2013). Serum contains more than thirty proteins of the complement system, and is a crucial component of the host innate immune response which can also initiate the adaptive response. The ability to inhibit complement activation is also considered a virulence trait. However, besides few examples of pathogenic bacteria that carry “*ad hoc*” molecules to block the activity of the complement (Kunert et al., 2007; Miajlovic and Smith, 2014), very little is known about the molecular basis of increased serum resistance and the clinical ramifications overall in *P. aeruginosa*.

We performed the present work using a morbidostat. A morbidostat is a semi-automated culturing device that continuously monitors bacterial growth and adjusts antibiotic concentration to induce bacterial resistance against the drug. In combination with “omic” approaches, experiments using a morbidostat can shed light on the different evolutionary trajectories of antibiotic resistance development as well as further phenotypic modification of clinical relevance. Our previous work with this device concluded that selection for colistin resistance results in a rise of mutations in *pmrB* and *pmrE* in the clinical strain *P. aeruginosa* PA77 (Dößelmann et al., 2017), which are common in other clinical isolates. Here we used the same clinical strain, derived from bloodstream infections and cultured it in our morbidostat for 21 days, under three experimental conditions: no antibiotic, single drug (colistin or metronidazole), and a combination of the two antibiotics. At four key time-points we measured the impact of antibiotic exposure on clinically relevant phenotypes such as antibiotic resistance, biofilm formation, serum resistance and virulence. We expect that the addition of metronidazole would lead to an alternate transcriptomic and phenotypic profile when compared to single drug treatment isolates. This is the first study to examine the evolution of an extensively drug resistant (XDR) *P. aeruginosa* strain in a morbidostat device under strong antibiotic pressure, and to demonstrate that the acquisition of colistin tolerance and resistance can affect phenotypic traits generally associated with virulence.

## Materials and Methods

### Strain Selection: Characteristics of PA77

The *P. aeruginosa* clinical strain PA77 was isolated at the University Hospital Tübingen, Germany (Willmann et al., 2014). PA77 is extensively drug resistant (XDR) (Magiorakos et al., 2012), being non-susceptible to all antibiotics except colistin and fosfomycin. Multilocus sequence typing analysis show PA77 belongs to the high-risk sequence type (ST) ST308 (Willmann et al., 2014).

### Study Design and Experimental Conditions

The morbidostat system was built following the detailed instructions by Toprak et al. (2012) with the modifications outlined in previous work (Dößelmann et al., 2017). As part of our hygiene protocol, we ran 80% ethanol through the tubing system and 3% sodium hypochlorite for 30 min each, and distilled water was used to rinse the tubing after each solution. The biological waste from the morbidostat was fed into a container with a neutralizing solution.

**FIGURE 1:**
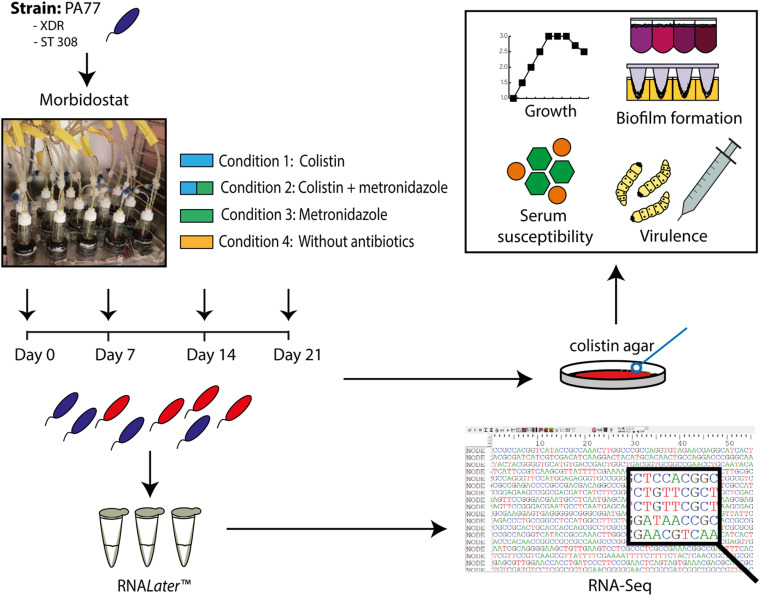
Experimental set up presented as a flow chart. The XDR strain PA77, ST 308, was cultivated in the morbidostat in 20 ml LB medium under four drug conditions: 2–500 mg/L colistin only (Condition 1), a combination of 2–500 mg/L colistin and 50 mg/L metronidazole (Condition 2) and 50 mg/L metronidazole only (Condition 3). A control condition without antibiotics was also completed (Condition 4). Samples of the isolates were taken every 2–3 days, with Day 0, 7, 14, and 21 selected as the key time-points of interest for our study. Isolates generated from the morbidostat were stored in RNA*Later* Stabilization Reagent (Qiagen, Hilden, Germany) to preserve the integrity and genetic profile of the bacteria. Finally, 36 isolates were generated in culture: from four different drug conditions, with three isolates per condition and three experimental time-points. The baseline strain PA77 is considered the Day 0 isolate. XDR, extensively drug resistant; ST, sequencing type. Blue isolates: susceptible to colistin (≤2 mg/L). Red isolates: colistin-resistant (>2 mg/L).

Figure 1 demonstrates an overview of the study design. Strain PA77 was continuously cultivated for 21 days in the morbidostat in four conditions. The first condition was with colistin as a single drug starting with 2 mg/L and with a final concentration of 500 mg/L. The second single drug condition is with 50 mg/L of metronidazole, and the third condition is with a combination of increasing colistin and 50 mg/L metronidazole. The fourth condition was completed as a control, with plain LB medium and no antibiotics. For every condition we ran three replicates in different vials in the morbidostat to investigate whether evolutionary trajectories were stable in all strains. We took samples of the culture at 7, 14, and 21 days of drug exposure, which means collectively there were three replicates over three time points, in four conditions. This totals 37 strains including the baseline strain of PA77 being the original clinical strain not evolved in the morbidostat. All strains were assessed in phenotypic and transcriptomics assays. These samples were processed with RNA*Later* Stabilization Reagent (Qiagen, Hilden, Germany) and ultimately frozen *via* the Microbank system (Pro-Lab Diagnostics Inc., TX, United States) at −80°C. All phenotypic assays were performed with one bead taken from these frozen stocks and grown overnight on blood agar plates containing 2 or 16 mg/L colistin. This was to ensure a truly resistant population, devoid of persister cells or dormant bacteria.

### DNA Extraction, Library Preparation and Genome Sequencing for Baseline Strain PA77

The genome sequencing was performed at the NGS Competence Center Tübingen (NCCT) using both short (Illumina) and long Oxford Nanopore Technology (ONT) reads for the baseline strain PA77. For ONT, genomic DNA was isolated using Qiagen Genomic Tip G/20, the integrity and quality of the preparations were evaluated on a Femto Pulse capillary electrophoresis device (Agilent, Santa Clara, CA, United States). Library preparation was performed with Ligation Sequencing Kit (SQK-LSK109) with Native Barcoding Expansion (NBD104). The pooled libraries were sequenced on a MinION flow cell (FLO-MIN106D) for 48 h. For short read sequencing, the DNA was extracted with the DNeasy UltraClean Microbial Kit (Qiagen). Illumina sequencing libraries were prepared using Nextera DNA Flex library prep kit (Illumina), using combinatorial dual indexing. The Illumina libraries were pooled and sequenced on a NextSeq 500 High-Output flow cell with 2 × 150 bp.

### *De novo* Genome Assembly and Annotation for Baseline Strain PA77

Whole genome assembly of the baseline strain was performed using the hybrid mode of Unicycler v.048 (Wick et al., 2017). Standard Unicycler parameters were used.

### *RNA*Later Storage

Samples were taken from the morbidostat every 2–3 days and stored in *RNA*Later RNA Stabilization Reagent (Qiagen, Hilden, Germany) to preserve the integrity and genetic profile of the isolates. Up to 10^8^ cells were centrifuged at 12,000 × *g* for 2 min. The supernatant was removed and the pellet resuspended in 1 ml of *RNA*Later solution and incubated for 1 h at room temperature. The pellet was centrifuged once more at 12,000 × *g* for 5 min, the supernatant removed and the remaining pellet was frozen at −80°C.

### RNA Sequencing

Total RNA was extracted with the Quick RNA Fungal/Bacterial Kit (Zymo Research), using lysozyme for cell lysis (0.4 mg/ml). The integrity and quality of the RNA preparations was evaluated with agarose gel electrophoresis and Agilent Bioanalyzer 2100 assays. Total RNAseq Library Kit (Zymo Research) was used for both ribosomal RNA depletion and RNAseq library preparation, using 500 ng total RNA as input. We constructed three libraries per RNA sample. The libraries were sequenced on a NextSeq 500 High-Output flow cell (75 cycles, single reads).

### Colistin Susceptibility of PA77 and Isolates Cultivated in Morbidostat

After comparison against broth microdilution (BMD) and other commercial tests with positive results (Javed et al., 2018), antibiotic susceptibility testing was determined using MICRONAUT MIC-Strip (MERLIN Diagnostika Gmbh, Bornheim, Germany): a commercial broth microdilution system using the international reference methodology (ISO 20776-1) and completed according to the manufacturer’s instructions. Briefly, isolates were taken from −80°C stocks and grown on plain agar plates overnight. An inoculum of 0.5 McFarland was prepared in 5 ml NaCl, and then further diluted 1:200 in Mueller Hinton broth. 100 μl of this dilution was pipetted into MIC-Strips which contained freeze dried colistin ranging from 0.0625 to 64 mg/L. The strips were incubated for 18–24 h at 37°C, and results read visually.

### Growth Assays

Isolates were grown overnight on selective colistin blood agar plates containing 2 or 16 mg/L colistin. A subculture was made to optical density (OD_600 nm_) 0.1 in LB medium. 200 μl of this subculture was transferred to sterile, flat-bottomed, polystyrene 96-well microtiter plates. The growth rate of bacteria was determined by incubating the plate at 37°C, measuring the OD of the culture in each well at 600 nm at 30 min intervals for 24 h using a microtiter plate reader (SPECTRAmaxPLUS384 Molecular Devices Inc., United States), and growth curves generated.

### Biofilm Formation Assay With Peg Lid Device

Isolates from −80°C storage were grown overnight on sheep blood agar plates containing 2 or 16 mg/L of colistin. After dilution of this culture to 0.5 McFarland in LB medium, 200 μl was transferred to all but the negative control wells of a flat-bottom 96-well microtiter plate (Nalgene Nunc International, Rochester, NY, United States) Polystyrene microtiter lids were immersed into this subculture (Nalgene Nunc International, Rochester, NY, United States) and incubated aerobically at 37°C for 2 h, followed by incubation at 37°C for 20–22 h anaerobically with Anaerocult tabs (Merck & Co., NJ, United States). The peg lids were dipped in 0.9% NaCl three times for 10 s each to remove planktonic bacteria, and sonicated in an ultrasonic bath (Sonorex^TM^ RK100) for 10 min. The peg lid is then placed in a mixture of 75 μl CHAPS and 75 μl EDTA solution on a rocking table (20 Hz) for 1 h, and the number of viable CFU per ml was determined by serial dilution plating, and counting colonies after 20–24 h incubation at 37°C.

### Biofilm Density Quantification With Crystal Violet Staining

Isolates from −80°C storage were grown overnight on sheep blood agar plates containing 2 or 16 mg/L of colistin. After dilution of this culture to 0.5 McFarland in LB medium, 200 μl was transferred to all but the negative control wells of a flat-bottom 96-well microtiter plate (Nalgene Nunc International, Rochester, NY, United States) and incubated aerobically at 37°C for 24 h. Planktonic cells were stained with an aqueous solution of 0.1% crystal violet for 30 min. The excess crystal violet was discarded, and wells were rinsed with distilled water. Stained biofilms were resuspended in 5% acetic acid for 30 min, and absorbance was measured at 590 nm by a microtiter plate reader. Assays were performed in triplicate in three independent experiments, with standard deviations indicated.

### Serum Selection

Normal human serum (NHS) from five healthy donors (Department of Transfusion Medicine, University Hospital Tübingen) was stored in aliquots at −80°C. The optimum serum dilution was determined for the baseline strain PA77. Normal human serum (NHS) from healthy donors (Department of Transfusion Medicine, University Hospital Tübingen) was stored in aliquots at −80°C. Strain PA77 was incubated with a percentage of serum, ranging from 10 to 90%. Serum was diluted with PBS (Gibco, Gaithersburg, MD, United States). They were then incubated with a luciferase compound at 37°C for 0, 2, and 4 h and the luminescence of ATP produced in culture measured as an indication of growth. It was determined that 50% was the optimum concentration of serum to use for this study.

### Serum Killing Assay

Serum killing assays were completed with BacTiter-Glo^TM^ Microbial Cell Viability Assay (Promega, Madison, WI, United States) as described (Necchi et al., 2017). Normal human serum (NHS) from healthy donors (Department of Transfusion Medicine, University Hospital Tübingen) was stored in aliquots at −80°C. Heat inactivated serum (HIS) was generated by incubating the serum at 56°C for 30 min.

Overnight culture of bacteria was diluted to OD 600 nm 0.1 and subcultured for 1 h. Strains were incubated at 37°C in 100 μl HIS- or NHS-PBS in a 96 well V-bottom microtiter plate (Greiner bio-one, Frickenhausen, Germany) in triplicates for 0, 2, 4, and 6 h. After incubation, plates were centrifuged at 3,500 × *g* for 5 min and the pelleted bacteria were resuspended in 100 μl PBS. To determine the number of viable bacterial cells, 50 μl bacterial suspension and 50 μl BacTiter-Glo^TM^ reagent were transferred to a white LUMITRAC^TM^ 96 well F-bottom microtiter plate (Greiner bio-one, Frickenhausen, Germany) and the adenosine triphosphate (ATP) levels produced by the bacteria were quantified with a Tecan Infinite^®^ 200 PRO. Resulting luminescence values were log transformed (natural logarithm) and the linear regression coefficients of the resulting growth curves in serum (log luminescence over time) were used to calculate a coefficient difference (CD). Here, the regression coefficient of the growth in HIS (αHIS) was subtracted from the regression coefficient of growth in NHS (αNHS). CD = αNHS – αHIS.

Growth in HIS was considered a growth control, hence the coefficient needed to be positive to be considered an adequate experimental setup. A CD value below 0 indicates either effective killing of bacteria by the serum or growth inhibition by the serum. Serum killing happened when the NHS coefficient was negative. Higher negative values would generally indicate a stronger impact of the serum on the bacteria, lower negative values indicate the development of resistance to serum. CD values ≥ 0 indicates no effect of the serum on growth, meaning a resistance to serum.

### *In vivo* Virulence Assays

*Galleria mellonella* larvae of the same size and weight range were purchased from Biosystems Technology (TruLarv^TM^). Subcultured bacteria were serially diluted in PBS to 8–10 CFU. Each *G. mellonella* larvae was injected with 10 μl of 8–10 CFU bacterial dilution using a 30 gage syringe (BD Biosciences, Allschwil, Switzerland). The larvae were then incubated at 37°C and monitored for 36 h after infection, and the death events were recorded every 2 h. Death was defined as when the larvae stopped responding to touch. Ten microliter aliquots of the bacterial dilutions injected into the larvae were plated in triplicates on LB agar plate and the CFU was determined after overnight incubation to ensure that the injected inoculum was in the 8–10 CFU/10 μl range. A hazard ratio (HR) was calculated with a Cox proportional hazard model (using Stata version 12.1), factoring in the CFU injected into each larvae.

### Identification of Differentially Expressed Genes With Geneious Prime

We used Geneious Prime (version 2020.0.5) to analyze and visualize the transcriptomic sequencing data. Reads from morbidostat-generated isolates were pre-processed with Trimmomatics (Bolger et al., 2014) aligned to the reference sequence PA77 using Bowtie2 (Langmead and Salzberg, 2012). We used six replicates per isolate, with at least 10 mio high quality reads. Expression levels of each isolate were compared, and volcano plots generated with the plugin DESeq2 (Love et al., 2014). The genes of interest were filtered by selecting the ratio of significance to 25 and a log2 fold expression of ±4.

### Statistical Analysis

GraphPad PRISM 5 (GraphPad Software, San Diego, CA, United States) and Stata version 12.1 (Stat Corp., College Station, TX, United States) were used to perform null hypothesis testing. All error bars represent standard deviation. For each figure, the number of replicates and other information relevant for assessing the accuracy and precision of the measurements are included in the corresponding legend.

## Results

### Characteristics of PA77 and Morbidostat Generated Isolates From NGS

Hybrid assembly of the baseline strain PA77 resulted in two contigs with total length of 6.9 Mbp. The baseline strain was used as the template for differential gene expression analysis.

### No Changes in Growth Rates Between Baseline, Control and Experimental Isolates

**FIGURE 2:**
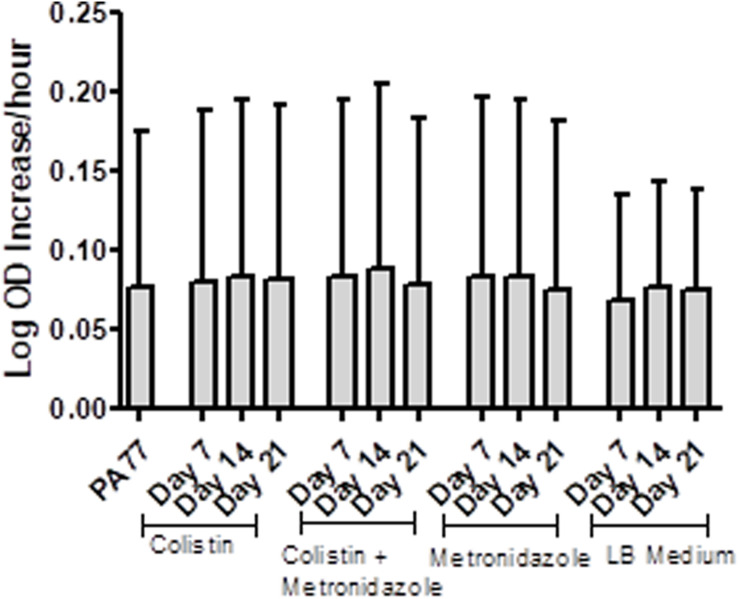
No changes in the growth rates of baseline strain and evolved isolates. Isolates were inoculated in plain LB medium and the optical density measured every 30 min at OD 600 nm, during incubation at 37°C for 24 h, with three replicates per isolate. The results are presented at Log OD increase per hour, with error bars representing the 95% confidence intervals.

Isolates from each condition and key time points were cultured in plain LB medium and growth measured every 30 min *via* OD reader. The results are displayed as Log-OD increase per hour (Figure 2) and as growth curves in Supplementary Figure 1. There was no difference in the log increase in OD between isolates, indicating that the growth kinetics of the evolved isolates were similar to those of the baseline strain.

### Resistance to Colistin Increases as Exposure in Morbidostat Continues for Up to 21 days

**FIGURE 3:**
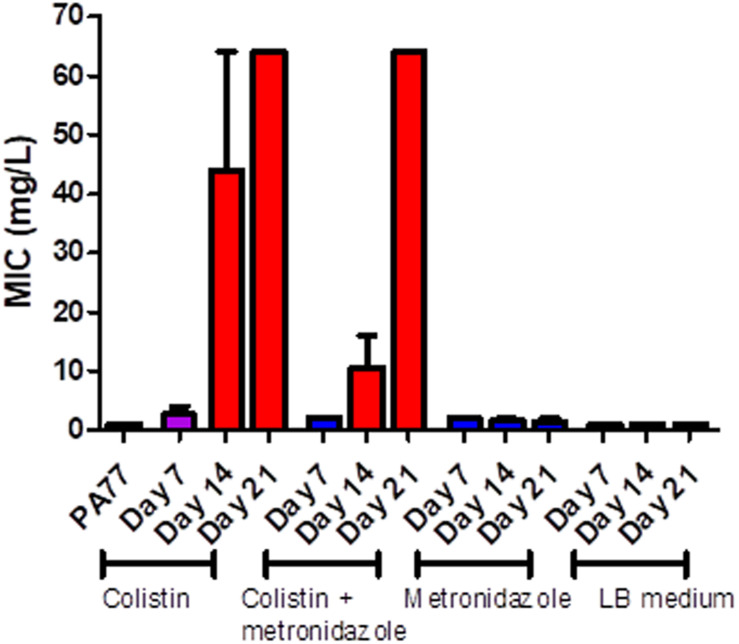
Measure of colistin sensitivity in baseline strain PA77 and evolved isolates. Distribution of the minimum inhibitory concentrations (MICs, mg/L) for colistin (*n* = 37) with isolates cultivated in the morbidostat across four different conditions and samples at three time-points: seven, 14 and 21 days of cultivation. The results are presented as the mean of three biological replicates and range. Blue: indicates susceptibility to colistin (MIC ≤ 2 mg/L). Red: indicates resistance to colistin (MIC > 2 mg/L). Purple: indicates a mix of replicates that are both susceptible and resistance to colistin.

PA77 strains continuously exposed to colistin reached a colistin MIC > 64 mg/L at Day 21. This is at least 16-fold higher than the clinical EUCAST breakpoint of colistin for *P. aeruginosa* at ≥2 mg/L (EUCAST, 2020; Figure 3). The metronidazole-only and LB medium control strains did not develop colistin resistance. The full MIC values are presented in Supplementary Table 1.

### Increase in Number of Viable Cells, Density and Biomass in Biofilm After Colistin Exposure

Next, we assessed whether the ability to form biofilms was altered under antibiotic exposure.

**FIGURE 4:**
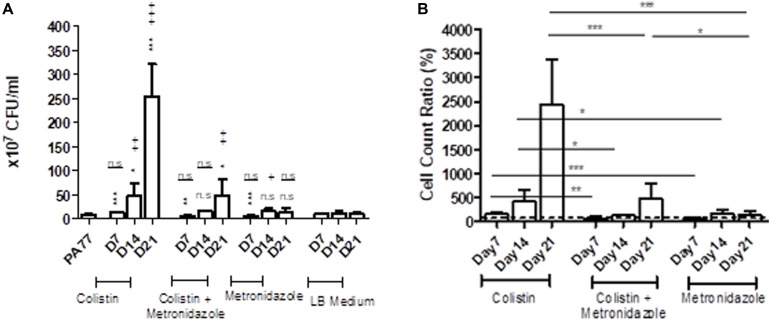
Altered number of viable cells in biofilms of the evolved isolates. **(A)** Measure of viable cells in biofilm produced by isolates cultivated in the morbidostat for 21 days in four different conditions: colistin only, a combination of colistin and metronidazole, metronidazole only and no antibiotic as a control condition. Six replicates per isolate were taken from the morbidostat and grown in LB medium for 24 h and the resulting biofilm detached chemically and mechanically, serially diluted and plated on agar. The CFU was counted and recorded as values from 10^7^. The statistical analysis shown are averages of at least three independent experiments ± sd. Comparison to the baseline strain PA77 is indicated by *n.s, not significant; *p < 0.05; **p < 0.01; ***p < 0.001. Comparison to LB medium isolates is denoted using +. n.s, not significant; +*p* < 0.05; ++*p* < 0.01; +++*p* < 0.001. **(B)** The values in panel A were adjusted to a ratio relative to LB medium isolates and displayed here as a bar chart, with a *t*-test used to calculate the difference between the three experimental conditions at each time point. *p < 0.05, ***p < 0.001. The dashed line represents the values for the control isolates, which is set to 100%.

Looking at the number of viable cells in biofilm, by far the biggest increase in development of biofilm occurred in the colistin only condition at Day 21, with strains producing 30-fold more viable cells in biofilm than the baseline strain (*p* < 0.001) (Figure 4A). All three time-points in this condition showed significantly increased biofilm production relative to the baseline. Looking deeper between drug conditions, we saw an increase in the number of viable cells in biofilm for isolates exposed to colistin for 7 days, with 14.5 × 10^7^ viable cells in biofilm compared to isolates exposed to metronidazole for the same time period with 6.7 × 10^7^ viable cells in biofilm (*p* < 0.001), and in the combination drug condition (6.1 × 10^7^ viable cells in biofilm) (*p* < 0.05) (Figure 4B).

Within 14 days of exposure to colistin as a single drug, there was a faster trajectory of increased biofilm formation than for isolates exposed to metronidazole only (*p* < 0.05) or in the combination of the two drugs (*p* < 0.05) (Figure 4B), which may indicate that metronidazole begins to affect the biofilm forming capabilities of the isolates. When compared to the baseline, there were a higher number of viable cells in biofilm in metronidazole-only isolates (17.5 × 10^7^) (*p* < 0.001) at Day 14 than the other conditions at this time-point (Figure 4A). After 21 days of exposure, isolates from the combination drug condition had fivefold more viable cells in biofilm than the baseline (*p* < 0.01), while the biofilm formed by metronidazole only isolates did not differ significantly. Between drug conditions, the metronidazole-only condition isolates showed 17-fold decrease in viable cells in biofilm after 21 days of exposure (14.7 × 10^7^ viable cells in biofilm) compared to colistin-only isolates at Day 21 (254.1 × 10^7^) (*p* < 0.001) (Figure 4B). The combination drug condition isolates also showed a slower trajectory of increased biofilm formation, with 80.5% less biofilm produced at Day 21 compared to the colistin-only condition (*p* < 0.001) (Figure 4B).

**FIGURE 5:**
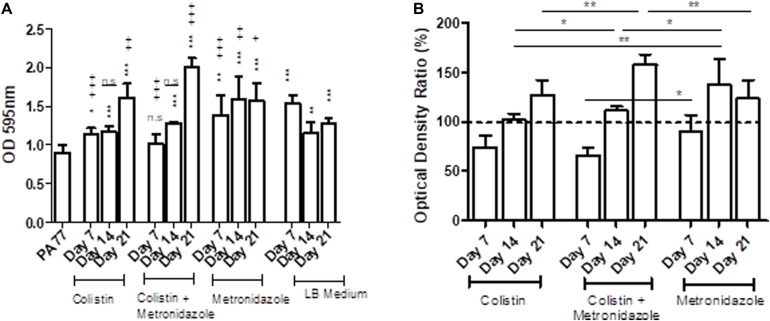
Changes in biomass in biofilms of the evolved isolates. **(A)** The OD values of biofilm staining assays using six replicates per isolate and the standard deviation are presented for strains cultivated in the morbidostat for 21 days in four different conditions: colistin only, a combination of colistin and metronidazole, metronidazole only and in LB medium as a control. The baseline strain PA77 was used as a Day 0 comparison. A *t*-test was used to compare the time-points to each other. Comparison to the baseline strain PA77 is indicated by *n.s, not significant; *p < 0.05; **p < 0.01; ***p < 0.001. Comparison to LB medium isolates is denoted using +. n.s, not significant; +*p* < 0.05; ++*p* < 0.01; +++*p* < 0.001. **(B)** The values of the experiments in panel A have been adjusted as a ratio, relative to the values of the LB medium isolates and displayed in a bar chart as percentages. The three experimental conditions have been compared according to the time point, and a *t*-test was used to calculate the significance. *p < 0.05, **p < 0.01, ***p < 0.001. The dashed line represents the values for the control isolates, and is set to 100%.

The metronidazole-only isolates showed a general increase in biomass in biofilm when compared to both the baseline strain and the control LB medium isolates (Figure 5A). The adjusted values showed isolates sampled at Day 7 in all three drug conditions show a decrease in biomass produced in biofilm compared to control isolates, with colistin-only isolates producing 76.2% of biomass in biofilm, and combination isolates producing 66.9% of biomass in biofilm. However, isolates cultivated in metronidazole only show an increase in biomass in biofilm compared to the two other drug conditions (Figure 5B). Isolates exposed to metronidazole for 14 days produced 26% more biomass in biofilm than isolates exposed to colistin and metronidazole at the same time point (*p* < 0.05) (Figure 5B). Isolates exposed to colistin for 14 days produced 36% less biomass in biofilm than isolates cultivated in metronidazole only (*p* < 0.01), and 10% less biomass in biofilm compared to combination drug isolates (*p* < 0.05). The isolates exposed to colistin and metronidazole for 21 days showed the highest increase in biomass in biofilm compared to the isolates cultured in the two other drug conditions, with 159% biomass measured, compared to 128.3% for colistin isolates (*p* < 0.01), and 124.6% biomass measured for metronidazole only isolates (*p* < 0.01).

### Isolates Become Resistant to Complement Factors in Serum Within 7 days of Colistin Exposure

**FIGURE 6:**
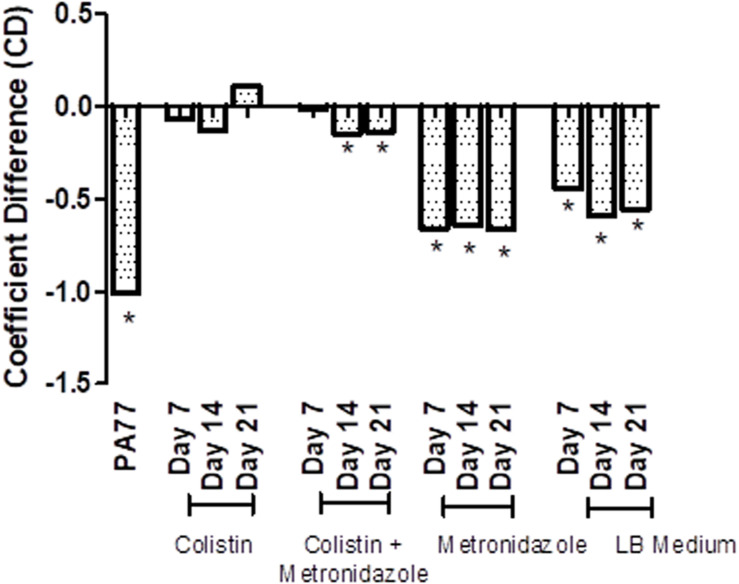
Transient decrease in susceptibility to serum in strains evolved in presence of colistin. Susceptibility to serum was measured by incubating *P. aeruginosa* isolates with 50% human serum and a luciferase compound, and measuring luminescence of ATP produced over 6 h. The results are presented as a bar graph with coefficient difference values calculated using log regression. Isolates with a negative αNHS value, indicating that they are killed in serum, are marked with an asterisk (*). PA77: baseline strain. Colistin: colistin only. Colistin + metronidazole: a combination of colistin and 50 mg/L metronidazole. Metronidazole: 50 mg/L metronidazole. LB medium: control condition isolates where isolates were not exposed to antibiotics and only cultured in LB medium.

Next, we tested resistance of the evolved isolates to complement factors (Figure 6 and Supplementary Figures 2A–M), with resistance being defined from the ability of these isolates to grow in human serum. The coefficient difference (CD) for the baseline strain is −1.01, which indicates a killing effect: the bacteria are not able to grow in 50% serum. However, after 7 days of exposure to colistin in the morbidostat, the isolates have a CD of −0.07, which indicates development of resistance to complement factors in the same serum. At Day 14 of colistin exposure, isolates were still resistant to serum complement, but less than at Day 7 (CD = −0.129). At Day 21, we see the biggest increase in serum resistance, with a CD of 0.108.

In the combination drug condition, there was also a notable development to serum resistance by Day 7 with a CD of −0.01. At Day 14 and Day 21, strains became again slightly more susceptible to serum (CD −0.148 and −0.139, respectively). The metronidazole-only and control condition do not show a strong deviation from the baseline susceptibility to serum.

### Decreased Virulence in *G. mellonella* After Infection With Isolates Exposed to Colistin for 21 days

**FIGURE 7:**
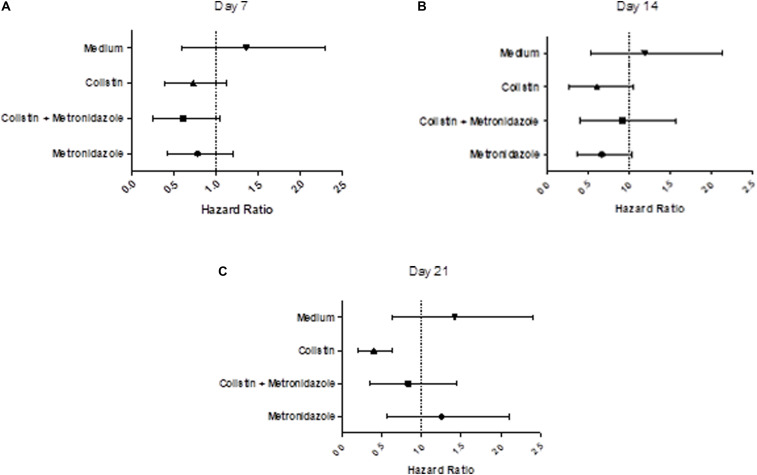
Prolonged exposure to colistin decreases virulence of *P. aeruginosa* PA77 in a *G. mellonella* model of infection. **(A–C)** The virulence potential of isolates was measured with *G. mellonella* larvae. The results are displayed as forest plots per time-point. The hazard ratio is calculated relative to the baseline condition strain PA77. Colistin (▲): colistin only. Colistin + metronidazole (■): a combination of colistin and 50 mg/L metronidazole. Metronidazole (●): 50 mg/L metronidazole. LB medium (▼): control condition isolates not exposed to antibiotics and only cultured in LB medium. **(A)** Day 7: Compared to the baseline strain, there is no significant change in virulence potential of these strains. **(B)** Day 14: There is no significant effect on virulence potential in any of these conditions overall, relative to the baseline strain. **(C)** Day 21: The isolates exposed to colistin in the single-drug condition show a decrease in virulence relative to the baseline strain.

We examined the virulence of the evolved isolates in a *G. mellonella* model of infection (Tsai et al., 2016; Figures 7A–C and Supplementary Figures 3A–C). Seven days of single exposure to colistin did not lead to a significant change in virulence (HR = 0.75, lower 95% CI: 0.40, upper 95% CI: 1.13, *p* = 0.13). At Day 14 we still did not observe a significant deviation from baseline. However, at Day 21 we noted a significant attenuation of virulence potential compared to the baseline (HR = 0.36, lower 95% CI: 0.20, upper 95% CI: 0.64, *p* = 0.01). This effect was not seen in the combination drug condition, with metronidazole seemingly having a modulatory effect on the impact of colistin exposure on virulence. Isolates cultured with metronidazole as a single-drug condition also demonstrated no statistically significant changes in virulence. This was the same for isolates in the control condition (LB medium only).

### Significant Variation in the Transcriptomic Profile of the Evolved Isolates Compared to Control Isolates

**FIGURE 8:**
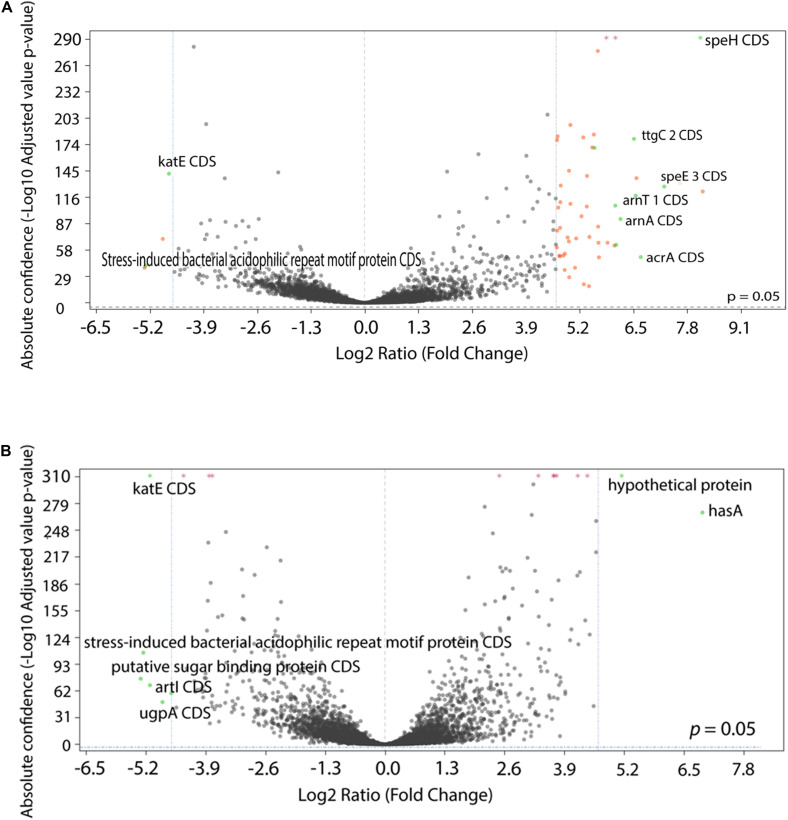
Comparison of differentially expressed genes between conditions. **(A,B)** The statistical significance and magnitude of change for the differentially expressed genes are depicted as volcano plots. Day 21 isolates are shown here, cultivated two conditions: **(A)** comparison between colistin only and baseline, and **(B)** comparison between control isolates grown in LB medium and baseline. The log2 fold change is plotted against the significance (–Log10 adjusted *p* value). The horizontal dashed line represents a ratio of significance, set at 0.05. Each point represents a gene, with genes of interest are highlighted in green and labeled. Genes that are within the significant interest threshold but unlabelled are orange. Genes that do not meet the criteria are colored in black.

To examine the transcriptional profile of the isolates that had been evolved under different drug pressures, we performed RNA-Seq analysis on *P. aeruginosa* PA77 at the baseline and on strains grown for 7, 14, and 21 days in presence of single or a combination of antibiotics. Due to the drastic changes in the phenotype of the morbidostat-generated strains, namely increased biofilm formation, decrease in virulence and loss of susceptibility to serum, we wanted to investigate general changes in the differentially expressed genes between the three time-points and four conditions (Figures 8A,B and Supplementary Figures 2A–J). For differential gene expression, a threshold of log2 fold change (FC) of ± 4 was applied.

Among the genes that were upregulated include the *arnBCADTEF* operon (also known as *pmrHFIJKLME*), which has a crucial role in colistin resistance in *P. aeruginosa* through the addition of 4-amino-4-deoxy-L-arabinose (L-Ara4N) to lipid A (Lo Sciuto et al., 2020). The *arnBCADTEF* operon is upregulated in colistin within 21 days (Figure 8A), but not differentially expressed at a significant level in isolates exposed to LB medium (Figure 8B) and metronidazole only (Supplementary Figure 2J).

Two other gene transcripts, *speH* and *speE*, were significantly upregulated in the colistin only and combination drug conditions, as part of the operon *speEH-pmrAB*. The highest positive log fold change for *speE* occurred in colistin Day 21 isolates with 7.25 (differential absolute confidence 128.25, *p* < 0.001) (Figure 8A) followed by colistin and combination isolates after 7 days of drug exposure: 6.83 (differential absolute confidence: 59.5, *p* < 0.001) and 5.95 (differential absolute confidence: 56.43, *p* < 0.001), respectively (Supplementary Table 2). In contrast, *speE* was downregulated in the medium-control isolates, with a log FC of −0.29 at Day 7 (differential absolute confidence: −1.96, *p* value: 0.006), −0.08 at Day 14 (differential absolute confidence: −0.06, *p* < 0.001) and a log fold change of 1.18 at Day 21 (differential absolute confidence: 5.9, *p* < 0.001) (Supplementary Table 2).

One gene of interest that was downregulated is *rpoS*. The highest fold change difference for this gene transcript in particular occurs after 7 days of exposure to metronidazole, with a downregulation in expression at −5.74 (differential absolute confidence −95.99, *p* < 0.001) (Supplementary Table 2), closely followed by isolates cultured in colistin for 14 days (log2 FC: −5.25, differential absolute confidence: −129.11, *p* < 0.001) and isolates cultivated in a mix of colistin and metronidazole for 7 days (log2 FC: −5.22, differential absolute confidence: −156.4, *p* < 0.001). Isolates in the medium-control condition fall below the threshold of ≤−4 log2 ratio FC, but are clearly downregulated none the less, ranging from log2 FC of −0.46 (LB medium Day 14) to −2.42 (LB medium Day 21) (Supplementary Table 2).

## Discussion

In this work, we used one XDR clinical strain of *P. aeruginosa* and cultured them in a morbidostat device with two antibiotics for 21 days, in order to discover the many ways the strain can evolve resistance in a simulated clinical setting. The aim of this work was to investigate the specific contribution of metronidazole alongside the development of colistin resistance in *P. aeruginosa* and evaluate the effect of antibiotic stress and on bacterial fitness, geno- and phenotype.

A number of assays were performed to elucidate the changes in the bacterial phenotype under distinct antibiotic pressure. Subinhibitory exposure of colistin has a clear effect in many of the factors that we have looked at: by the end of 21 days there was increased biofilm formation, a loss in susceptibility to serum and a decrease in virulence. Metronidazole seemingly contributes a modulatory effect on two of these factors: with less viable cells in biofilm and less susceptibility to serum complement factors in combination and metronidazole only condition isolates, as measured by quantifying the concentration of ATP in serum over time. Interestingly, isolates cultured in colistin, both as a single drug and in combination are unable to grow in either heat-inactivated serum or active serum at 6 h, unlike isolates cultivated in metronidazole, which continue to grow in heat-inactivated serum. This indicates a developed sensitivity to heat-stable factors in serum as a result of exposure to sub-inhibitory concentrations of colistin.

Analysis of the transcriptomic profile uncovered a series of differential gene expression patterns. One gene of interest is *speE*, encoding a spermidine synthase, an enzyme that catalyzes the irreversible transfer of a propylamine group from the amino donor *S*-adenosylmethioninamine (decarboxy-AdoMet) to putrescine (1,4-diaminobutane) to yield spermidine. Polyamines such as spermidine are involved in several biological processes, including binding to nucleic acids, stabilizing membranes, and protecting against host immune responses (Chou et al., 2008). Spermidine may be of clinical importance as a biofilm inhibitor: catheters immersed with norspermidine were effective in disrupting mature biofilm (Qu et al., 2016). Exogenous spermidine also protected *P. aeruginosa* against polymyxin B through stabilization of lipopolysaccharides (LPS), while endogenously synthesized spermidine conferred a protective effect against the host immune response to clinical strains of *P. aeruginosa* that produce high amounts of it (Johnson et al., 2012). Its upregulation as part of the operon *speEH-pmrAB* is interesting, as *pmrAB* genes are well established as mediating resistance to colistin and other antibiotics (Lee et al., 2005; Bricio-Moreno et al., 2018) with one study finding that the PmrAB regulon activates *speE* in the presence of antimicrobial peptides such as colistin (Bolard et al., 2019).

Our transcriptomics results indicated that exposure to increasing concentrations of colistin led to significant increased expression of *speH* and *speE* (*speE/H*). The upregulation of these genes may be a mechanism to confer tolerance to colistin under antibiotic pressure in the morbidostat, particularly as it is not highly differentially expressed in isolates exposed to metronidazole only and is found upregulated in isolates that are colistin-resistant. The efficacy of colistin is directly related to the LPS structure, specifically through binding to anionic LPS components of the outer membrane, causing increase of cell permeability and cell death by cell lysis. *P. aeruginosa* defends against colistin by the addition of l-4-aminoarabinose (l-Ara4N) to lipid A phosphates (Nowicki et al., 2014). The proteins for the synthesis and transfer of l-Ara4N are encoded by the *arnBCADTEF* operon and are regulated by the PmrAB and PhoPQ two-component regulatory systems. *In vitro* evolution studies confirm this effect: high-level colistin resistance does not evolve in the absence of a functional *arnBCADTEF* operon (Lo Sciuto et al., 2020).

Upregulation of the *arnBCADTEF* operon–as we have observed it under colistin exposure–results in decreased polymyxins binding to the cell surface and the development of cross-resistance to colistin and other antibiotics (Romano et al., 2019). The LPS is also an inducer of the complement system, activated *via* its components lipid A, core, and O-antigen. Bacteria expressing long O-antigen chains are usually more resistant to serum than their O antigen-deficient isogenic mutants (Hong and Payne, 1997; Burns and Hull, 1998; Bravo et al., 2008) and are particularly necessary for serum resistance in *P. aeruginosa* (Ohno et al., 1995). Naturally, there was no resistance to colistin for isolates exposed to only metronidazole; these isolates also did not become resistant to serum and the gene transcripts *speE/H* and the *arnBCADTEF* operon were not upregulated significantly (Supplementary Table 2). Resistance to complement is strongly associated with the capability of systemic survival, multiplication, and spread of a wide range of Gram-negative pathogens (Short et al., 2020). Therefore we speculate that modifications in the LPS *via* upregulation of the *speE/H* genes and *arnBCADTEF* operon, regulated by pmrAB, may contribute to colistin resistance and loss of susceptibility to serum in our colistin-resistant isolates generated in the morbidostat.

The results of the virulence assays completed using *G. mellonella* show a significant decrease in virulence for one type of isolate: those exposed to colistin for 21 days (Figure 7C and Supplementary Figure 3C). The remaining isolates over the four conditions and three time-points show no significant difference in virulence potential when compared to the baseline strain PA77. This effect has been found in another recent study using Gram negative bacteria (Esposito et al., 2018). This study that assessed the virulence profile of 16 colistin resistant *K. pneumoniae* isolates with different levels of colistin resistance, found that the colistin MIC of *K. pneumoniae* isolates is predictive of their lethality (LD_50_ and LD_90_ values) in *G. mellonella*. High colistin MIC values were predictive of lower virulence of the isolates, indicating that genetic adaption to high levels of colistin resistance could somehow impair *K. pneumoniae* infectivity, although they did not observe any significant correlation between colistin-resistance mechanisms and virulence. In other research, *P. aeruginosa* with LPS deficiencies display attenuation in virulence in *G. mellonella* (Jarrell’ and Kropinski, 1982; Tsai et al., 2016) and so it is likely that isolates exposed to colistin for 21 days may have undergone transcriptional changes related to LPS modifications that make them lose their virulence potential. Metronidazole alone as a single drug treatment did not have any effect on virulence. Isolates exposed to metronidazole in combination with colistin, tend to be relatively virulent when compared to the single drug isolates. This indicates that metronidazole slows down the trajectory of decreasing virulence for strains evolved in combination with colistin.

The downregulation of expression of the RNA polymerase-encoding gene *rpoS* in response to the drugs colistin and metronidazole is surprising, especially considering its role as a major stress-response regulator: it was expected that this gene would be upregulated in isolates cultured with antibiotics in the morbidostat. It is well documented that *rpoS* is expressed mainly during the stationary growth phase (Kojic and Venturi, 2001), and it may be that the continuous changes in growth kinetics in the morbidostat due to the addition of colistin at regular intervals may have led to an alternative transcriptomic profile. Alternatively, the reduced expression of *rpoS* in isolates exposed to colistin and metronidazole over 21 days, relative to the baseline strain PA77 might have been an adaptation mechanism to increase resistance to colistin. Indeed, previous studies have reported that *rpoS* mutants of *P. aeruginosa* produced more biofilm, and biofilms produced were much more resistant to being killed by tobramycin than wild-type *P. aeruginosa* biofilms (Whiteley et al., 2001). They also found enhanced flagellar motility exhibited by the *rpoS* mutants, which led to increase in biomass in biofilm, similar to what we have seen in our study. Increased and better developed biofilm protect against penetration of colistin (Ciofu and Tolker-Nielsen, 2019) with extracellular polymeric substances reported to be the greatest barrier to diffusion for drug penetration into various bacteria (Wei and Ma, 2013). We see more biofilm produced in isolates exposed to colistin, either as a single treatment or in combination, than with metronidazole only. As deletion of this transcription factor has several important downstream effects (Lombardo et al., 2004), its reduced expression is likely to contribute to the altered transcriptional profile observed in the evolved isolates. *RpoS* controls close to 800 genes (Schuster et al., 2004) including several virulence factors such as pyocyanin, exotoxin A, LasA and LasB elastases, and exoenzyme S, and affects the expression of genes related to quorum sensing. Some of these genes are downregulated in our morbidostat-generated isolates, such as heat shock protein *dnaK*, and chaperonins *groES* and *groEL*, although not to a significant level (Supplementary Table 2).

The semi-automated morbidostat provides several advantages over traditional serial transfer evolution experiments. It enables us to simulate a clinical situation, as it represents compartments of infection within the human body that have not been fully eradicated by antibiotic treatment. These compartments of infection are exposed to sub-lethal concentrations of antibiotic, contributing to general tolerance. Isolates cultured in the morbidostat received sub-inhibitory doses of colistin, which creates a constant level of antibiotic pressure. The addition of metronidazole, which has no bactericidal effect on *P. aeruginosa*, but is a documented trigger for mutagenesis, leads to a significantly varied phenotype. In cases where colistin resistance develops, virulence potential decreases. However, isolates are able to survive and grow in human serum longer than their colistin-sensitive counterparts. Alongside the ability to maintain a higher number of viable cells in biofilm, the results of our work indicate the potential of a change in the course of infection due to significant evolutionary events under antibiotic treatment.

## Data Availability Statement

The datasets presented in this study can be found in online repositories. The names of the repository/repositories and accession number(s) can be found below: ebi.ac.uk; PRJEB41763.

## Author Contributions

MW and MJ designed the initial study setup. MJ, BJ, MW, SS, and SP selected strains, designed, and wrote protocols for the phenotypic experiments. MJ, BJ, MH, and VU completed phenotypic experiments, with MJ and MW analysing resulting data. MJ, MH, and VU performed wet lab work for NGS sequencing. MJ, MW, and AA performed NGS data analysis. US performed statistical analysis for experiments. MJ wrote the initial manuscript. MW compiled the final manuscript. All authors contributed to the article and approved the submitted version.

## Conflict of Interest

The authors declare that the research was conducted in the absence of any commercial or financial relationships that could be construed as a potential conflict of interest.
