# Supplementary material for: Transcriptomic Basis of Serum Resistance and Virulence Related Traits in XDR P. aeruginosa Evolved Under Antibiotic Pressure in a Morbidostat Device
Source: Front Microbiol. 2021 Jan 25;11:619542. doi: 10.3389/fmicb.2020.619542 (PMC7868568; doi:10.3389/fmicb.2020.619542)
Supplement: Supplementary file 1 [file Data_Sheet_1.zip › Supplementary_Frontiers/Supplementary_Figure_1.docx]

Supplementary Material

**Supplementary Figure S1: Growth curves of PA77 and evolved isolates in LB medium over 24hrs**. Isolates were diluted to OD 0.1 and incubated at 37°C for 24 hours. Experiments were performed in triplicates; the mean values are shown here with the standard deviation.

##
