# Supplementary material for: Transcriptomic Basis of Serum Resistance and Virulence Related Traits in XDR P. aeruginosa Evolved Under Antibiotic Pressure in a Morbidostat Device
Source: Front Microbiol. 2021 Jan 25;11:619542. doi: 10.3389/fmicb.2020.619542 (PMC7868568; doi:10.3389/fmicb.2020.619542)
Supplement: Supplementary file 1 [file Data_Sheet_1.zip › Supplementary_Frontiers/Supplementary_Figure_2.pptx]

## Slide 1
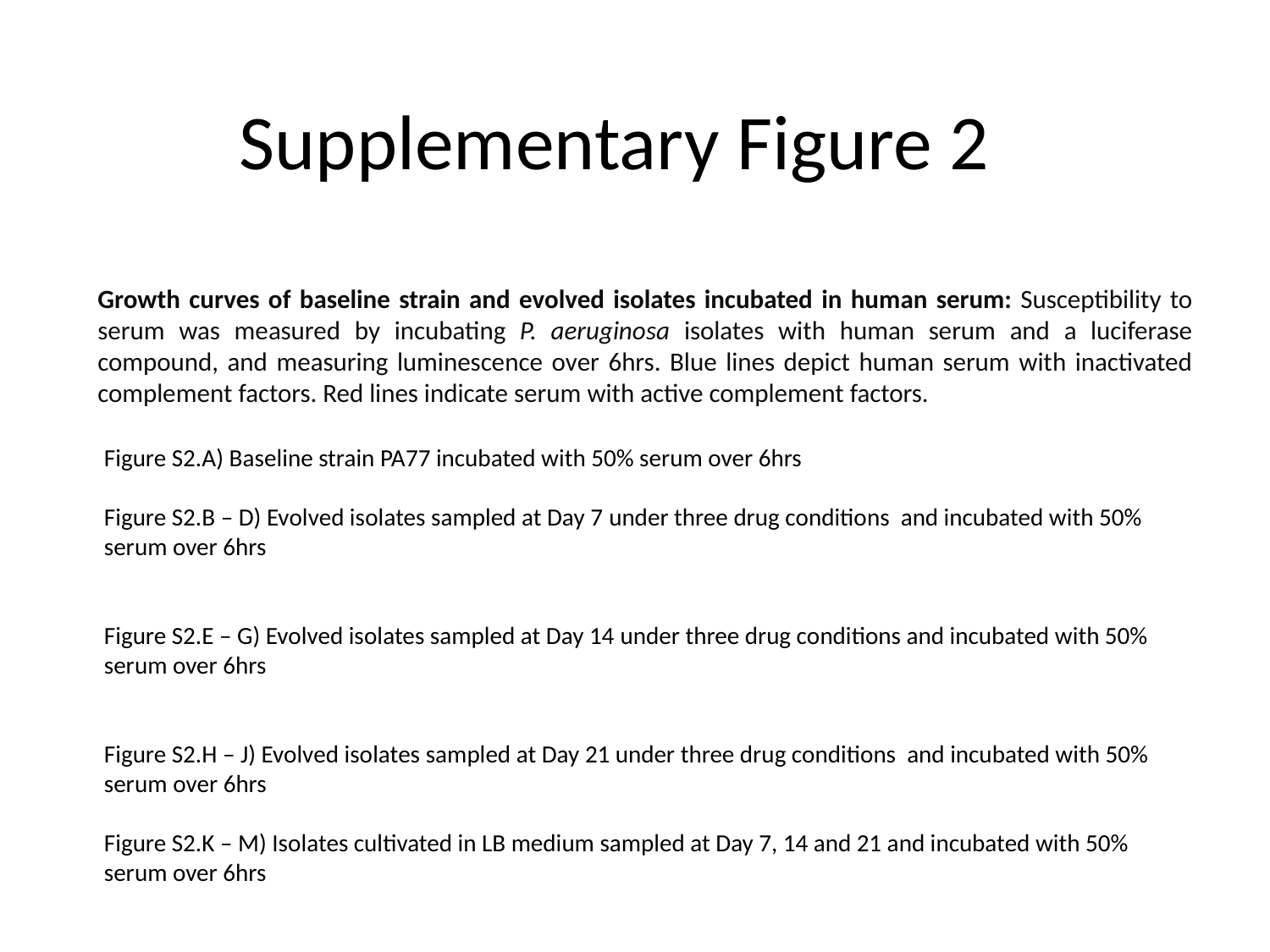

# Supplementary Figure 2
Growth curves of baseline strain and evolved isolates incubated in human serum: Susceptibility to serum was measured by incubating P. aeruginosa isolates with human serum and a luciferase compound, and measuring luminescence over 6hrs. Blue lines depict human serum with inactivated complement factors. Red lines indicate serum with active complement factors.
Figure S2.A) Baseline strain PA77 incubated with 50% serum over 6hrs
Figure S2.B – D) Evolved isolates sampled at Day 7 under three drug conditions and incubated with 50% serum over 6hrs
Figure S2.E – G) Evolved isolates sampled at Day 14 under three drug conditions and incubated with 50% serum over 6hrs
Figure S2.H – J) Evolved isolates sampled at Day 21 under three drug conditions and incubated with 50% serum over 6hrs
Figure S2.K – M) Isolates cultivated in LB medium sampled at Day 7, 14 and 21 and incubated with 50% serum over 6hrs

## Slide 2
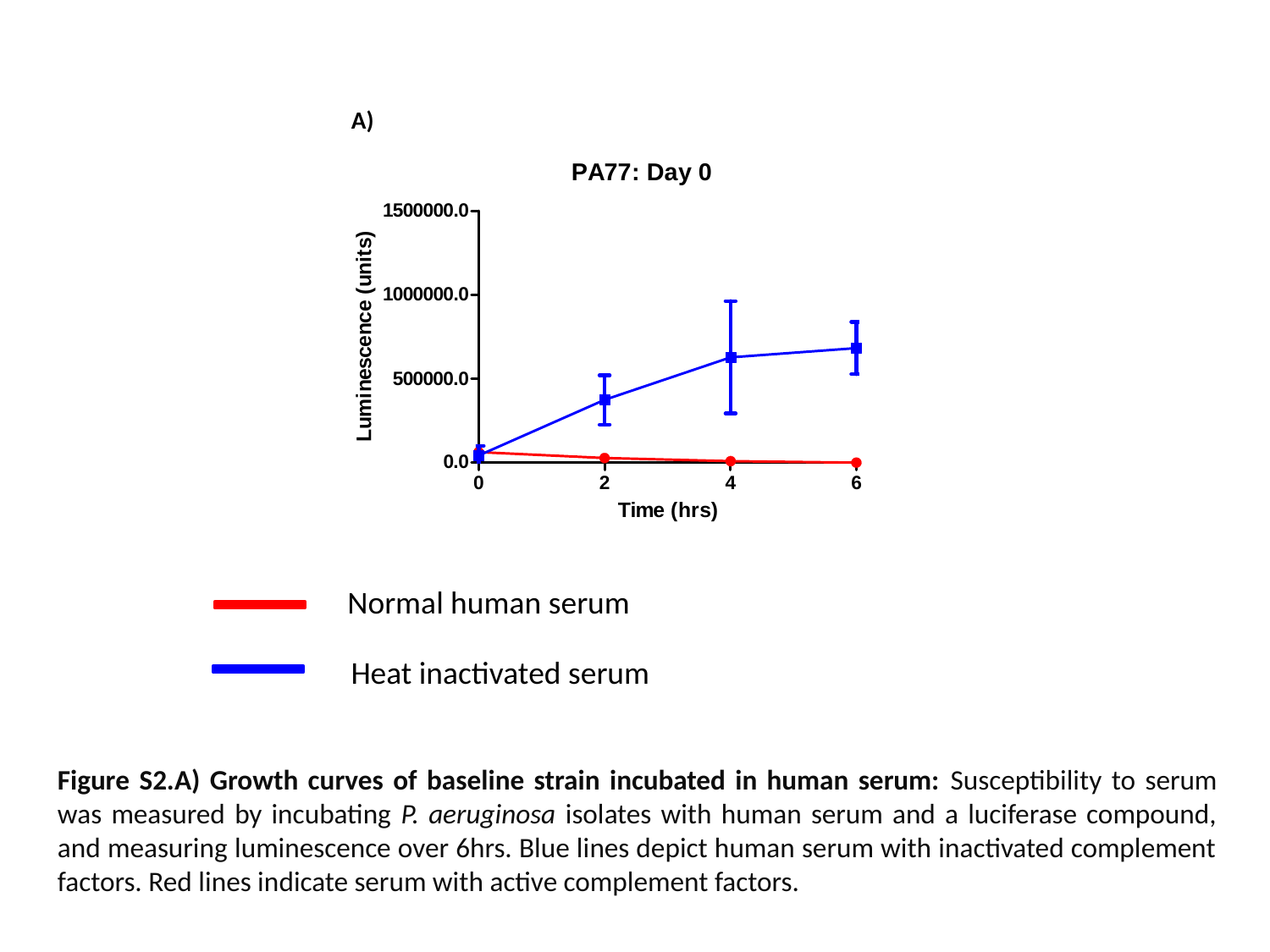

A)
Normal human serum
Heat inactivated serum
Figure S2.A) Growth curves of baseline strain incubated in human serum: Susceptibility to serum was measured by incubating P. aeruginosa isolates with human serum and a luciferase compound, and measuring luminescence over 6hrs. Blue lines depict human serum with inactivated complement factors. Red lines indicate serum with active complement factors.

## Slide 3
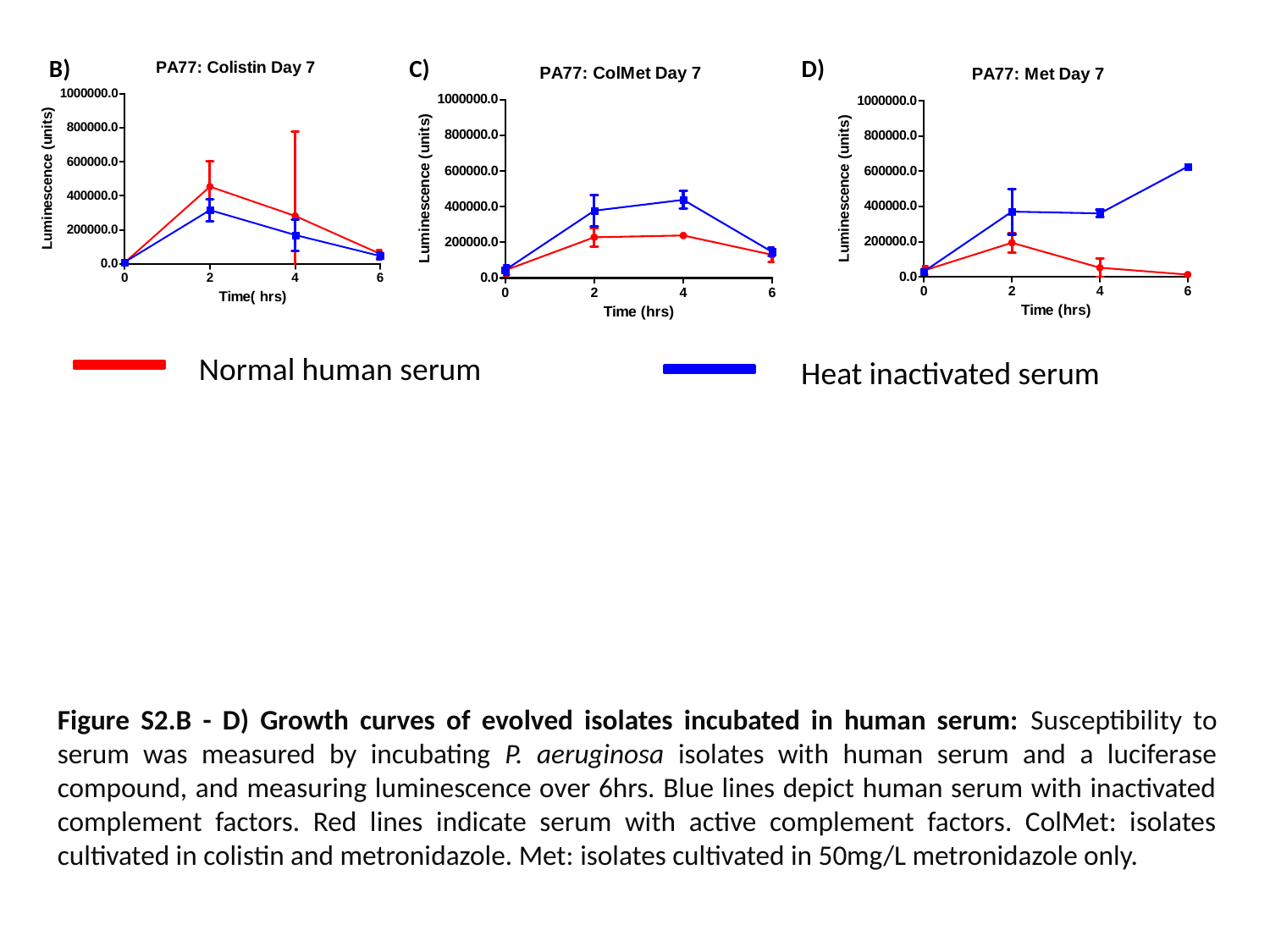

C)
B)
D)
Normal human serum
Heat inactivated serum
Figure S2.B - D) Growth curves of evolved isolates incubated in human serum: Susceptibility to serum was measured by incubating P. aeruginosa isolates with human serum and a luciferase compound, and measuring luminescence over 6hrs. Blue lines depict human serum with inactivated complement factors. Red lines indicate serum with active complement factors. ColMet: isolates cultivated in colistin and metronidazole. Met: isolates cultivated in 50mg/L metronidazole only.

## Slide 4
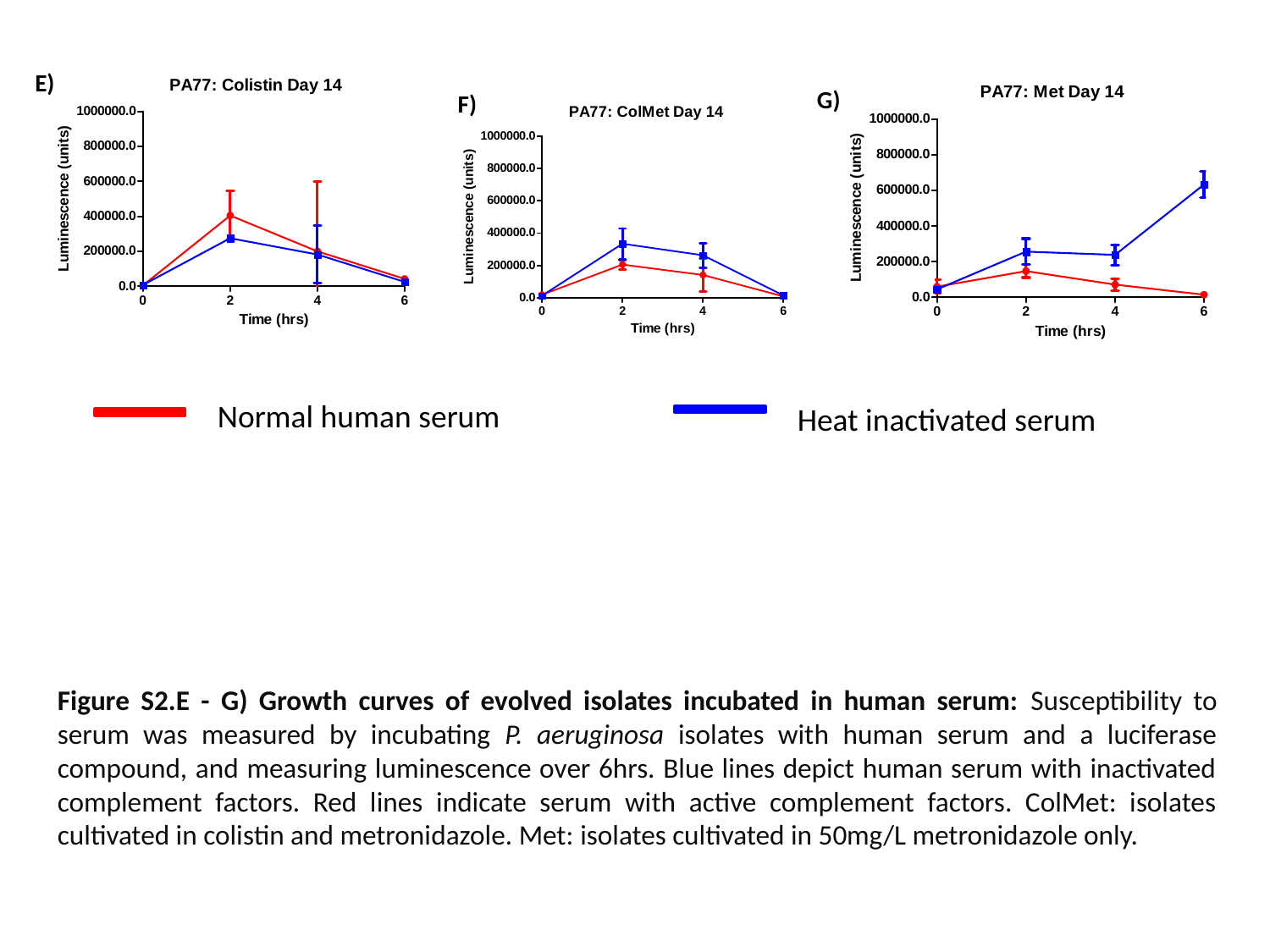

E)
G)
F)
Normal human serum
Heat inactivated serum
Figure S2.E - G) Growth curves of evolved isolates incubated in human serum: Susceptibility to serum was measured by incubating P. aeruginosa isolates with human serum and a luciferase compound, and measuring luminescence over 6hrs. Blue lines depict human serum with inactivated complement factors. Red lines indicate serum with active complement factors. ColMet: isolates cultivated in colistin and metronidazole. Met: isolates cultivated in 50mg/L metronidazole only.

## Slide 5
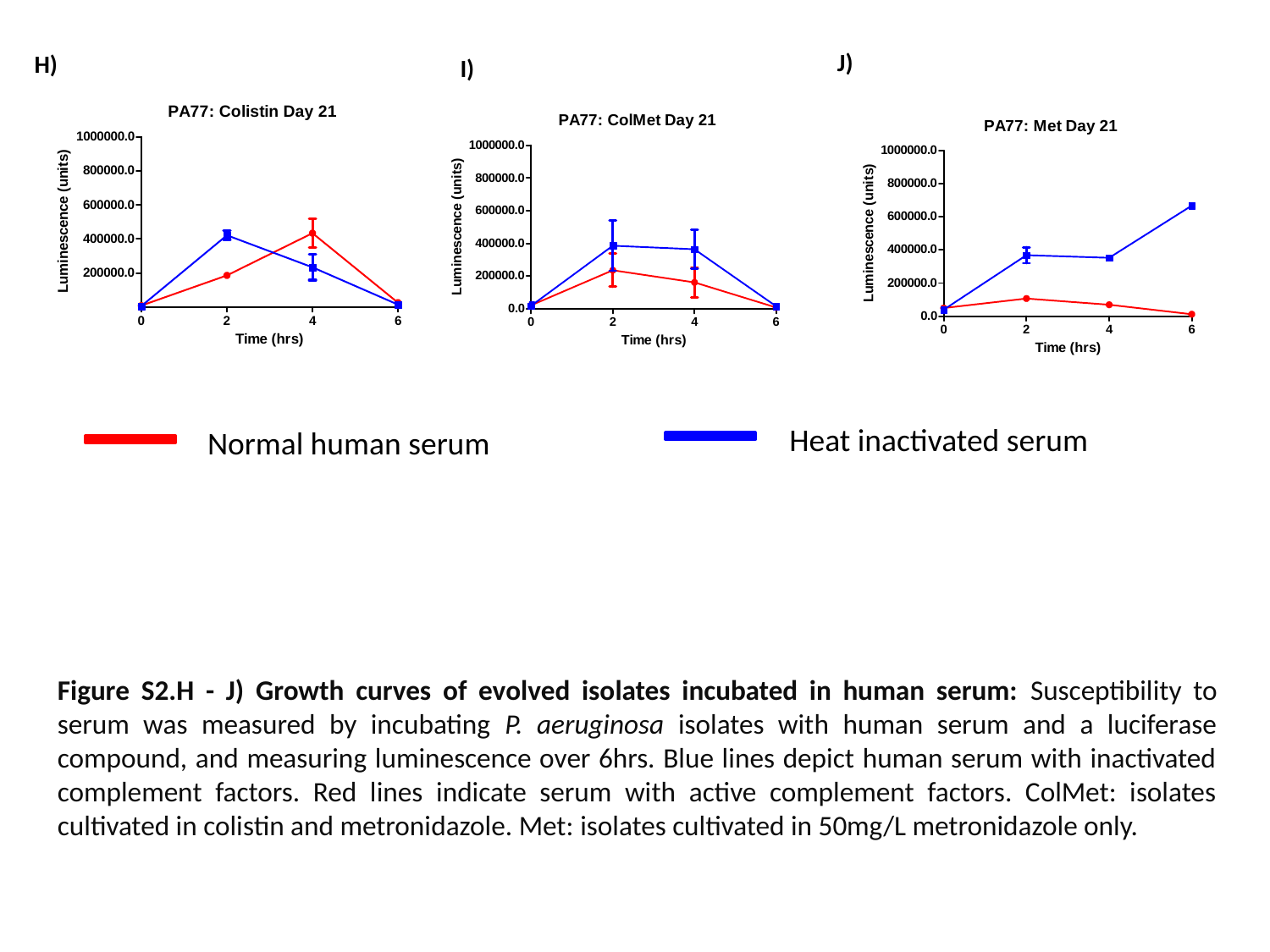

J)
H)
I)
Heat inactivated serum
Normal human serum
Figure S2.H - J) Growth curves of evolved isolates incubated in human serum: Susceptibility to serum was measured by incubating P. aeruginosa isolates with human serum and a luciferase compound, and measuring luminescence over 6hrs. Blue lines depict human serum with inactivated complement factors. Red lines indicate serum with active complement factors. ColMet: isolates cultivated in colistin and metronidazole. Met: isolates cultivated in 50mg/L metronidazole only.

## Slide 6
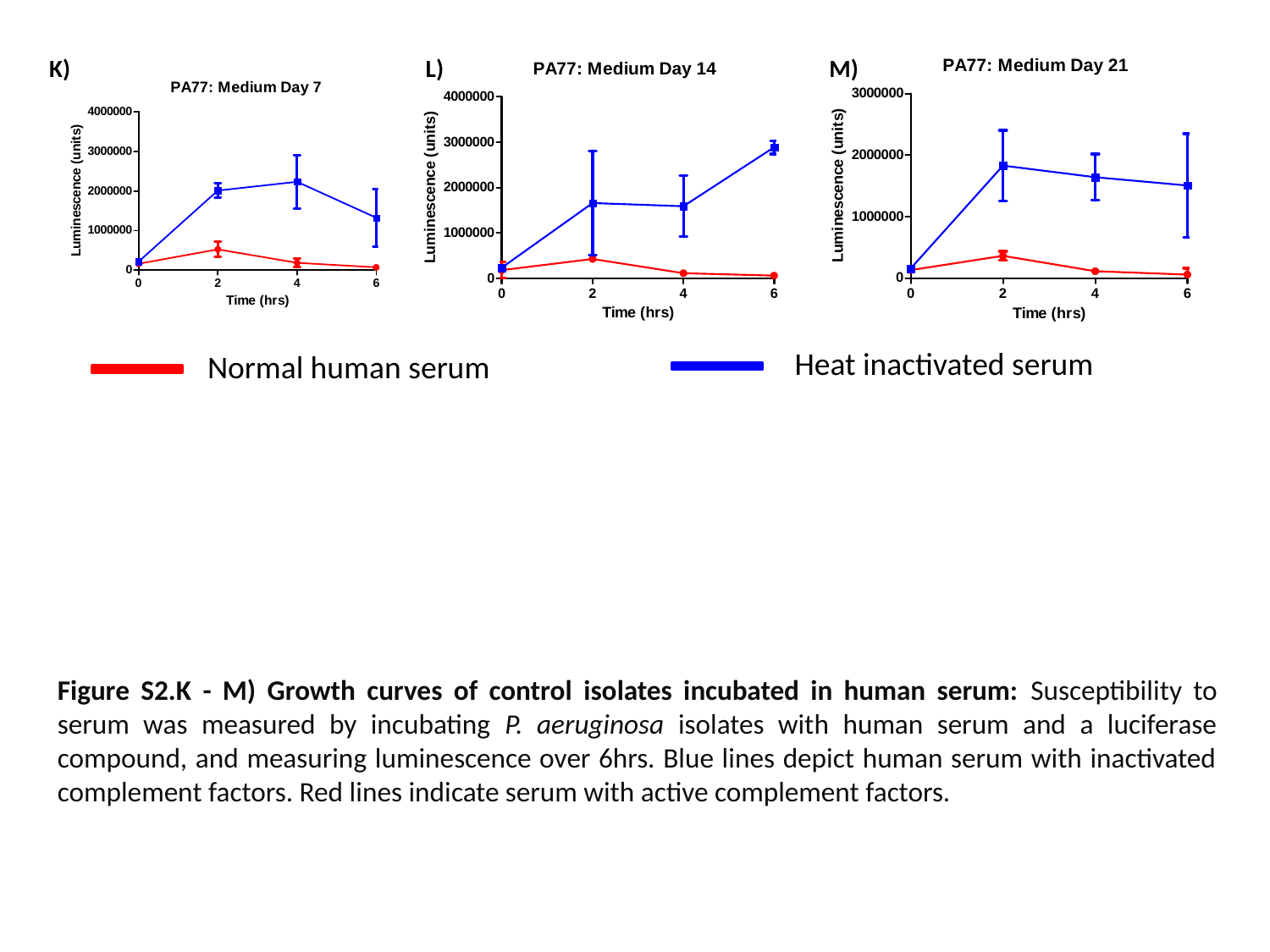

M)
L)
K)
Heat inactivated serum
Normal human serum
Figure S2.K - M) Growth curves of control isolates incubated in human serum: Susceptibility to serum was measured by incubating P. aeruginosa isolates with human serum and a luciferase compound, and measuring luminescence over 6hrs. Blue lines depict human serum with inactivated complement factors. Red lines indicate serum with active complement factors.
