# Supplementary material for: Transcriptomic Basis of Serum Resistance and Virulence Related Traits in XDR P. aeruginosa Evolved Under Antibiotic Pressure in a Morbidostat Device
Source: Front Microbiol. 2021 Jan 25;11:619542. doi: 10.3389/fmicb.2020.619542 (PMC7868568; doi:10.3389/fmicb.2020.619542)

Supplementary Material

**Supplementary Figure 3:** The virulence potential of isolates was measured with *G. mellonella* larvae. The results are displayed as Kaplan-Meier survival curves. Subcultured bacteria were serially diluted in PBS to 8-10 CFU and injected into each *G. mellonella* larvae, with 30 replicates in total. The death events were counted over 36hrs. **(A)** Kaplan-Meier survival curves using PA77 and isolates cultivated in colistin, a combination of colistin and metronidazole, metronidazole and LB medium for seven days. **(B)** Kaplan-Meier survival curves using PA77 and isolates cultivated in colistin, a combination of colistin and metronidazole, metronidazole and LB medium for 14 days. **(C)** Kaplan-Meier survival curves using PA77 and isolates cultivated in colistin, a combination of colistin and metronidazole, metronidazole and LB medium for 21 days.

**(A)**


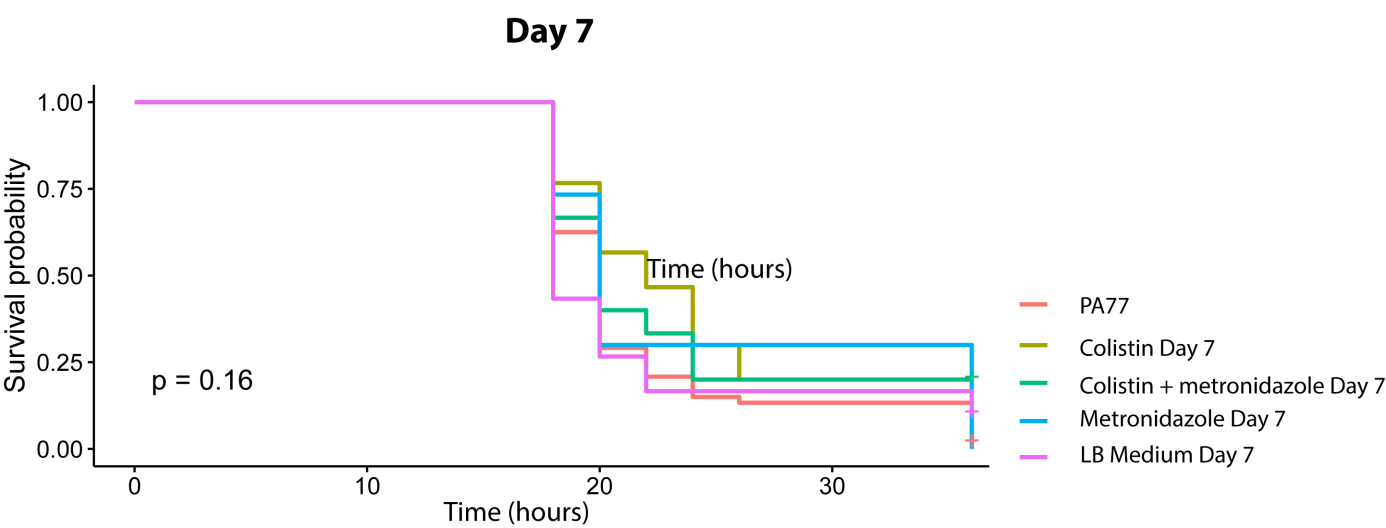


**(B)**


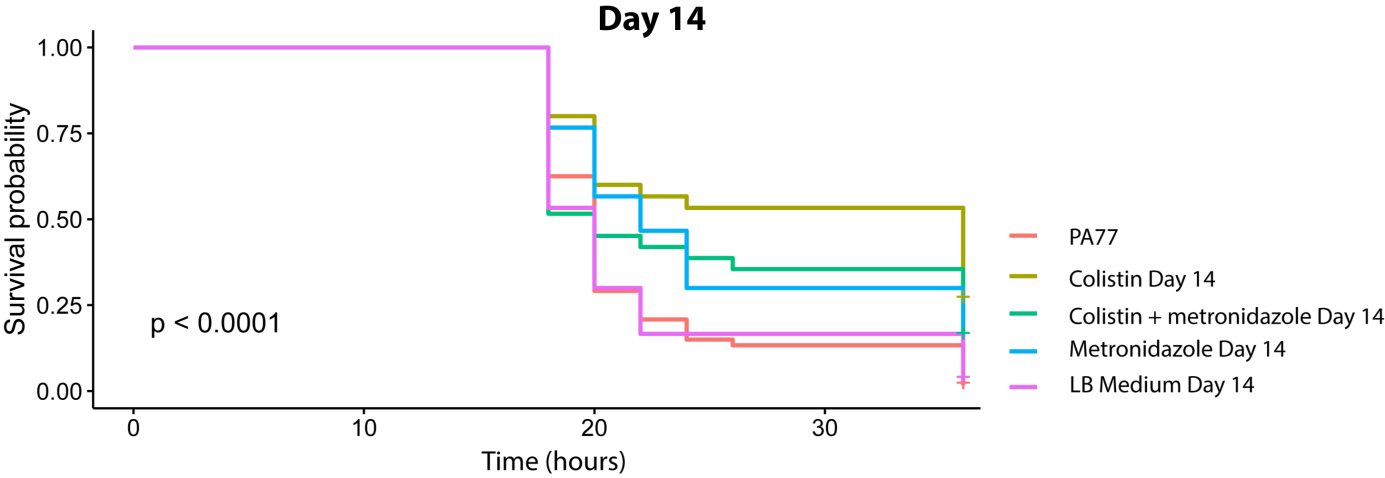


**(C)**

##
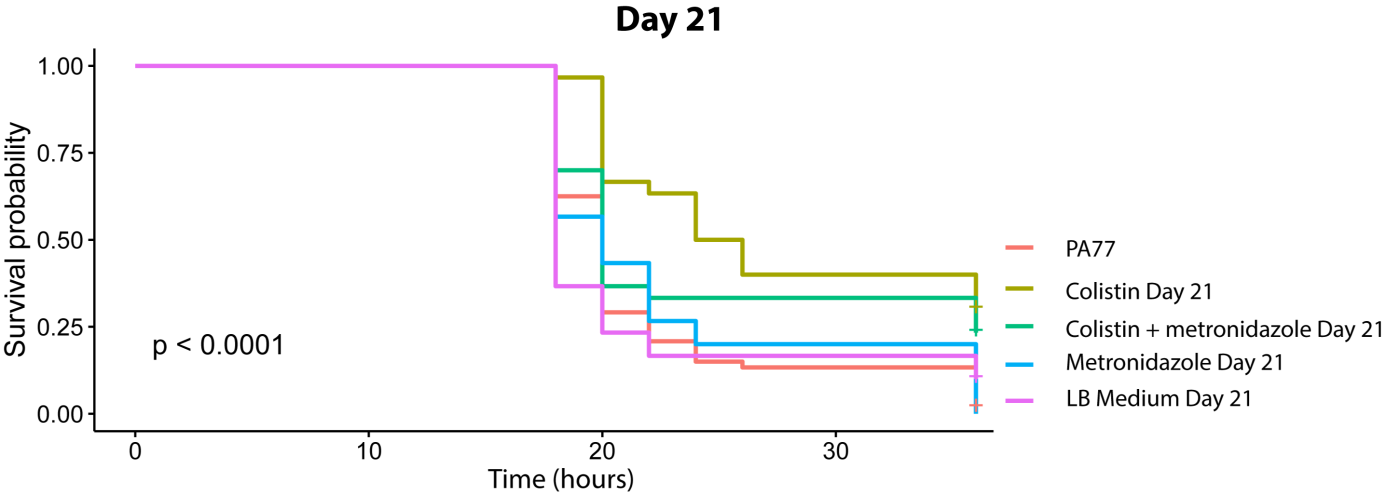

Supplement: Supplementary file 1 [file Data_Sheet_1.zip › Supplementary_Frontiers/Supplementary_Figure_3.docx]
