# Supplementary material for: Transcriptomic Basis of Serum Resistance and Virulence Related Traits in XDR P. aeruginosa Evolved Under Antibiotic Pressure in a Morbidostat Device
Source: Front Microbiol. 2021 Jan 25;11:619542. doi: 10.3389/fmicb.2020.619542 (PMC7868568; doi:10.3389/fmicb.2020.619542)
Supplement: Supplementary file 1 [file Data_Sheet_1.zip › Supplementary_Frontiers/Supplementary_Figure_4.pptx]

## Slide 1
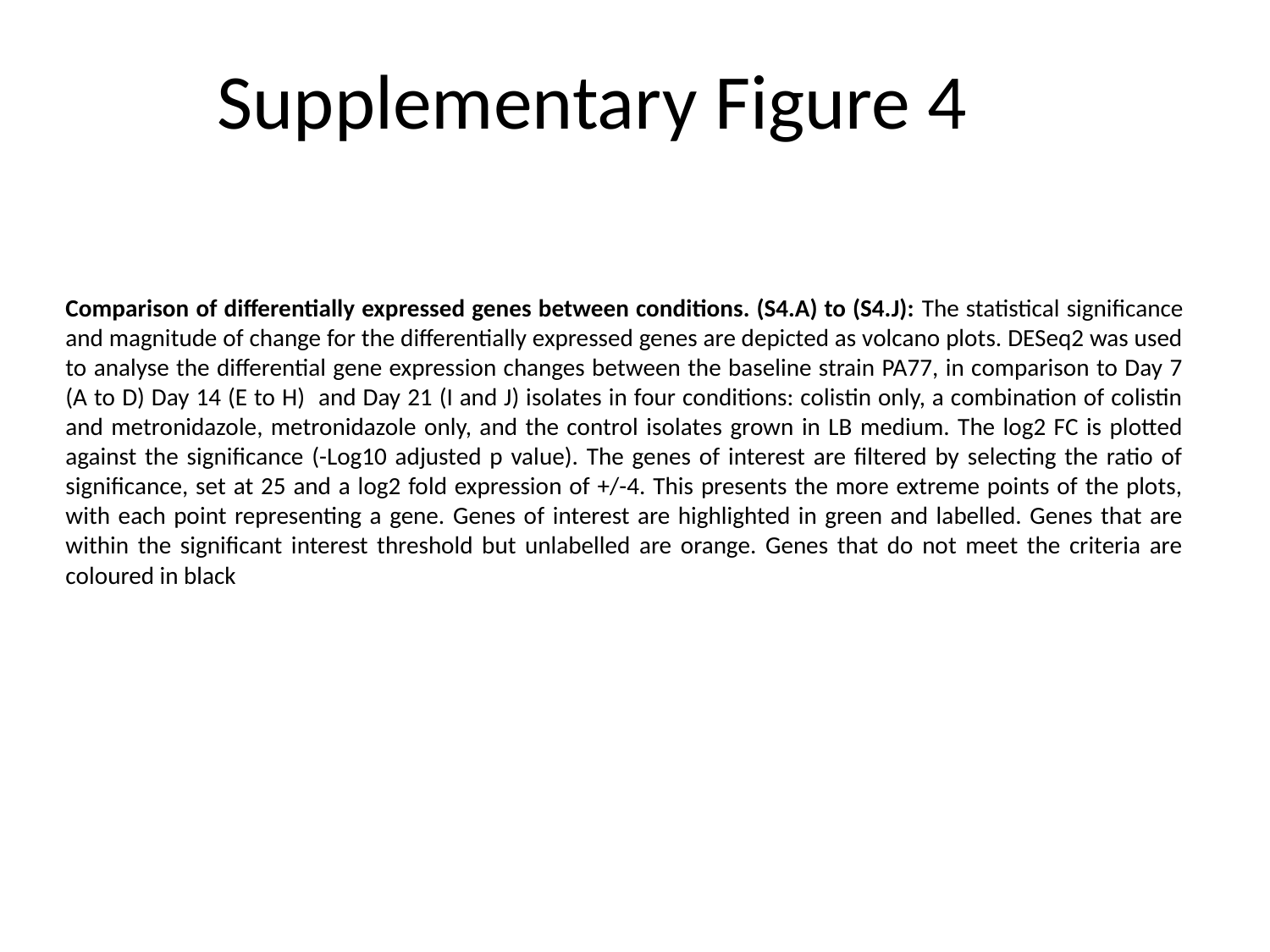

# Supplementary Figure 4
Comparison of differentially expressed genes between conditions. (S4.A) to (S4.J): The statistical significance and magnitude of change for the differentially expressed genes are depicted as volcano plots. DESeq2 was used to analyse the differential gene expression changes between the baseline strain PA77, in comparison to Day 7 (A to D) Day 14 (E to H) and Day 21 (I and J) isolates in four conditions: colistin only, a combination of colistin and metronidazole, metronidazole only, and the control isolates grown in LB medium. The log2 FC is plotted against the significance (-Log10 adjusted p value). The genes of interest are filtered by selecting the ratio of significance, set at 25 and a log2 fold expression of +/-4. This presents the more extreme points of the plots, with each point representing a gene. Genes of interest are highlighted in green and labelled. Genes that are within the significant interest threshold but unlabelled are orange. Genes that do not meet the criteria are coloured in black

## Slide 2
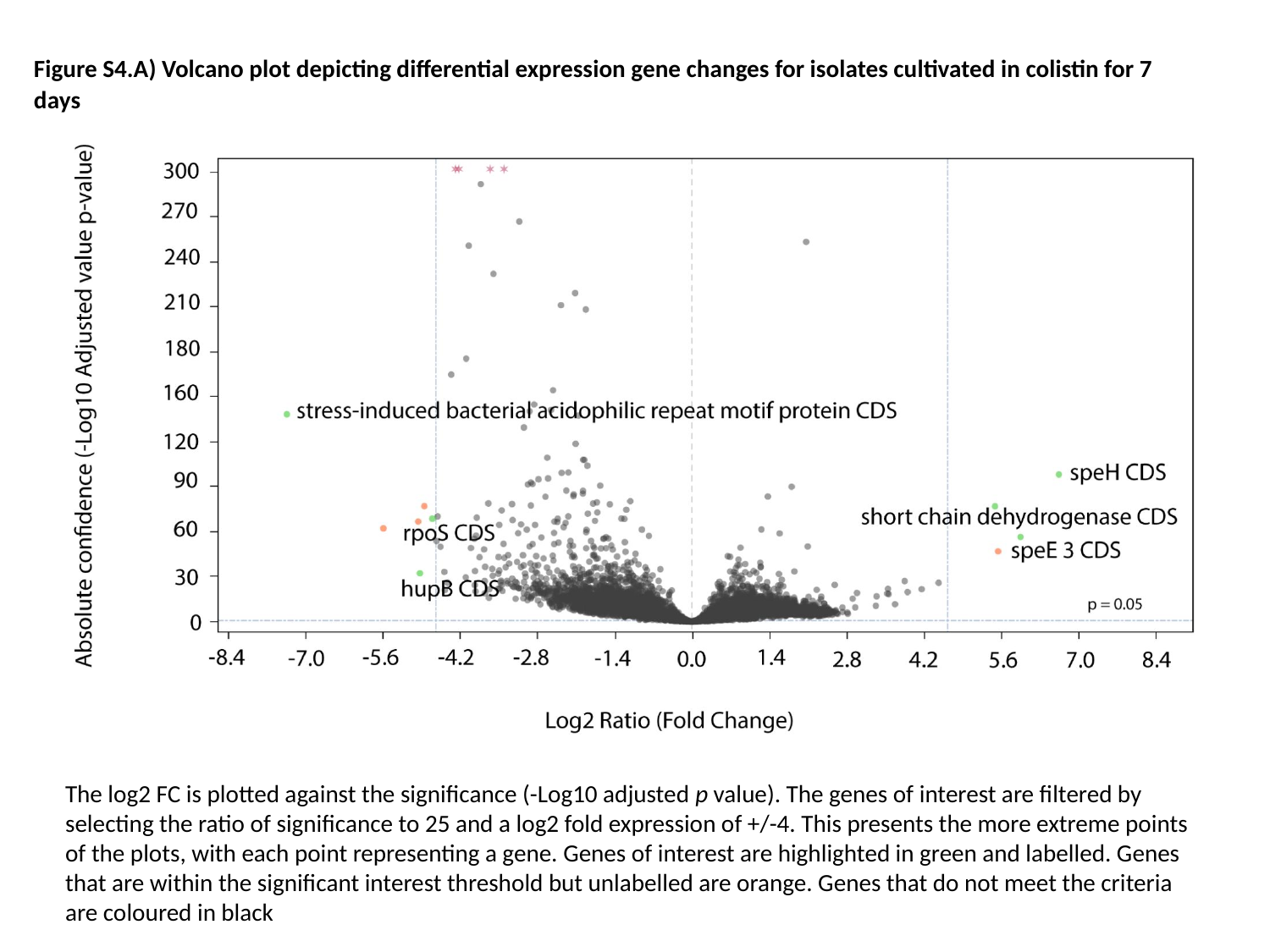

Figure S4.A) Volcano plot depicting differential expression gene changes for isolates cultivated in colistin for 7 days
The log2 FC is plotted against the significance (-Log10 adjusted p value). The genes of interest are filtered by selecting the ratio of significance to 25 and a log2 fold expression of +/-4. This presents the more extreme points of the plots, with each point representing a gene. Genes of interest are highlighted in green and labelled. Genes that are within the significant interest threshold but unlabelled are orange. Genes that do not meet the criteria are coloured in black

## Slide 3
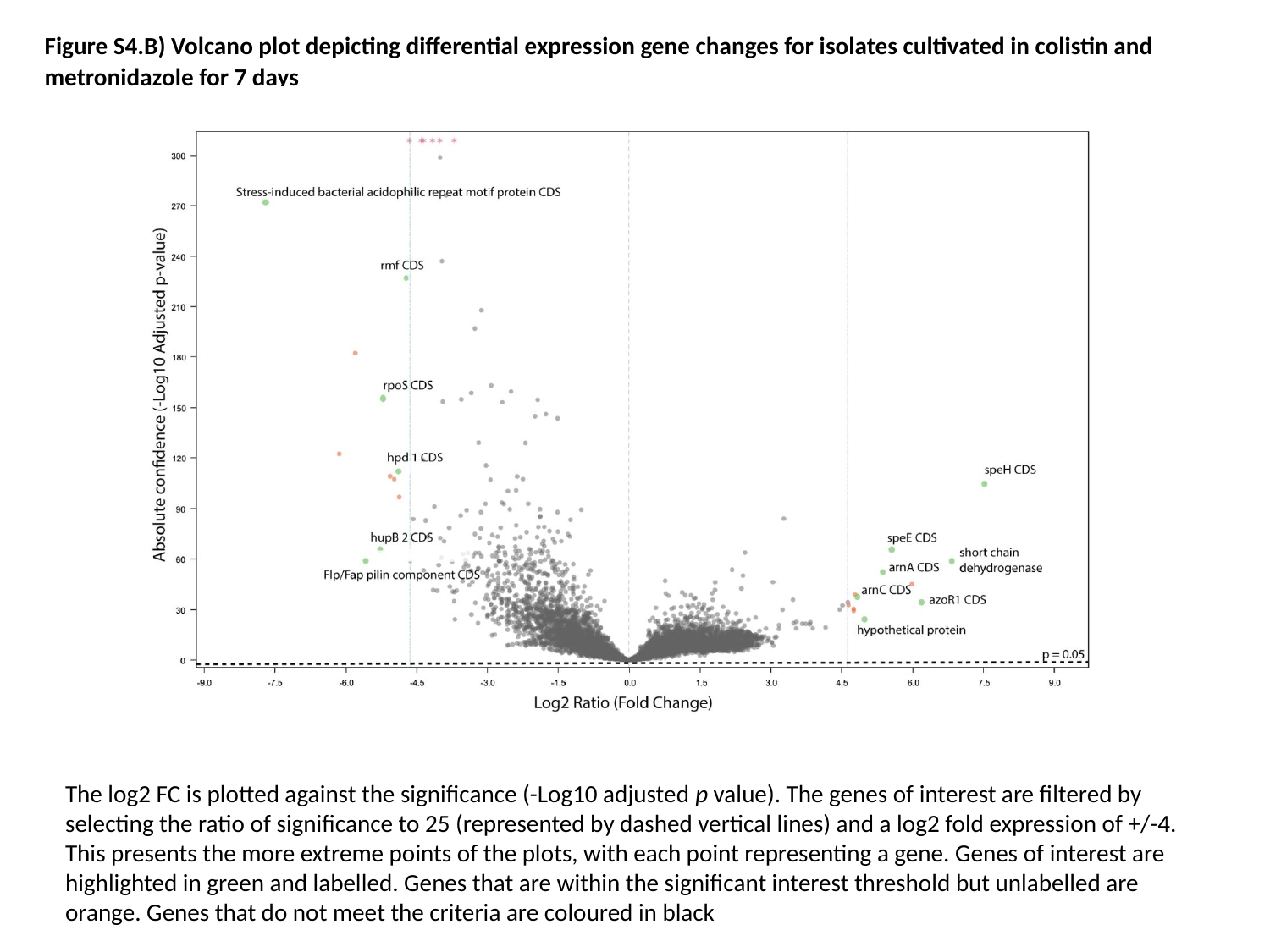

Figure S4.B) Volcano plot depicting differential expression gene changes for isolates cultivated in colistin and metronidazole for 7 days
The log2 FC is plotted against the significance (-Log10 adjusted p value). The genes of interest are filtered by selecting the ratio of significance to 25 (represented by dashed vertical lines) and a log2 fold expression of +/-4. This presents the more extreme points of the plots, with each point representing a gene. Genes of interest are highlighted in green and labelled. Genes that are within the significant interest threshold but unlabelled are orange. Genes that do not meet the criteria are coloured in black

## Slide 4
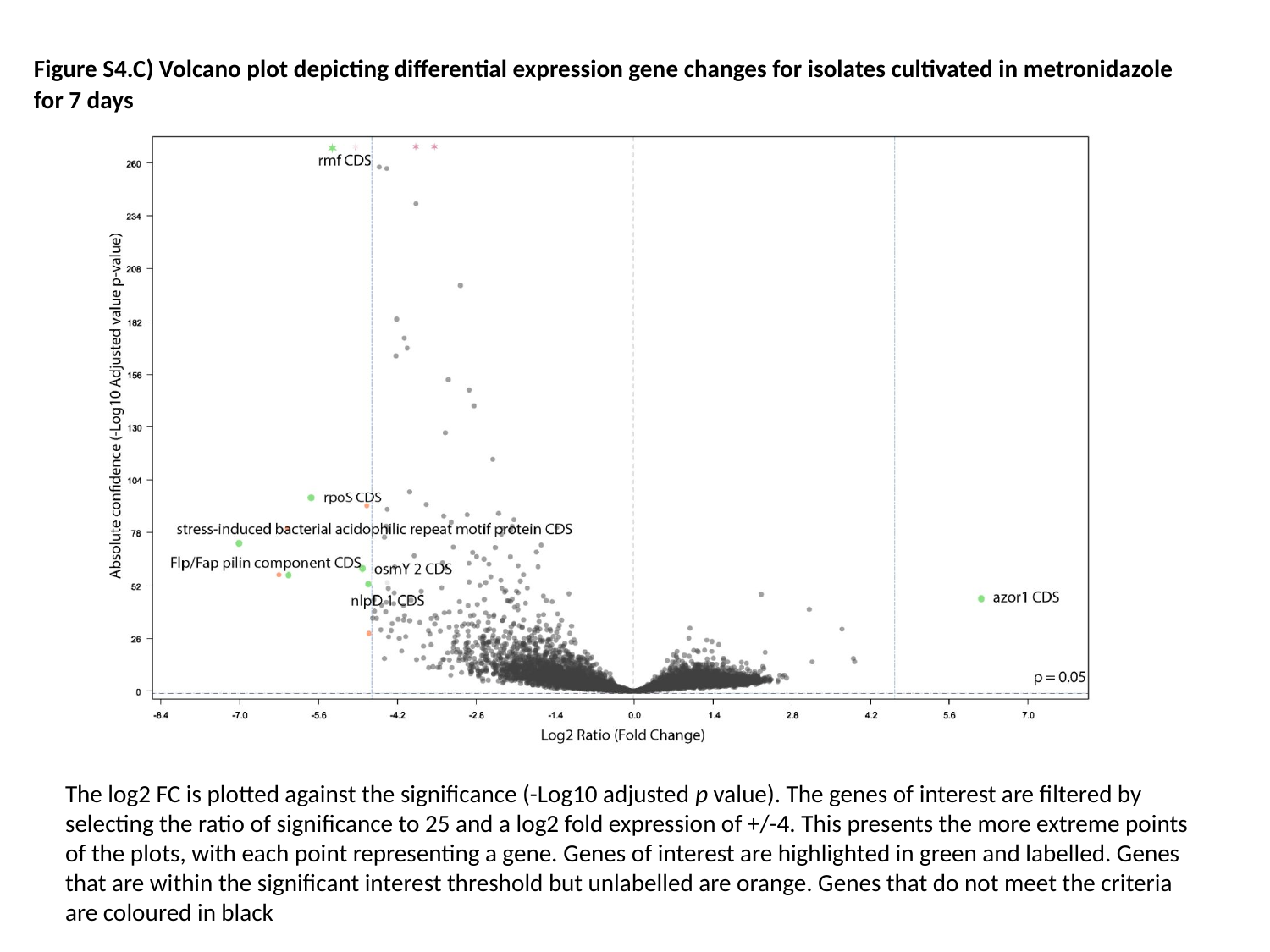

Figure S4.C) Volcano plot depicting differential expression gene changes for isolates cultivated in metronidazole for 7 days
The log2 FC is plotted against the significance (-Log10 adjusted p value). The genes of interest are filtered by selecting the ratio of significance to 25 and a log2 fold expression of +/-4. This presents the more extreme points of the plots, with each point representing a gene. Genes of interest are highlighted in green and labelled. Genes that are within the significant interest threshold but unlabelled are orange. Genes that do not meet the criteria are coloured in black

## Slide 5
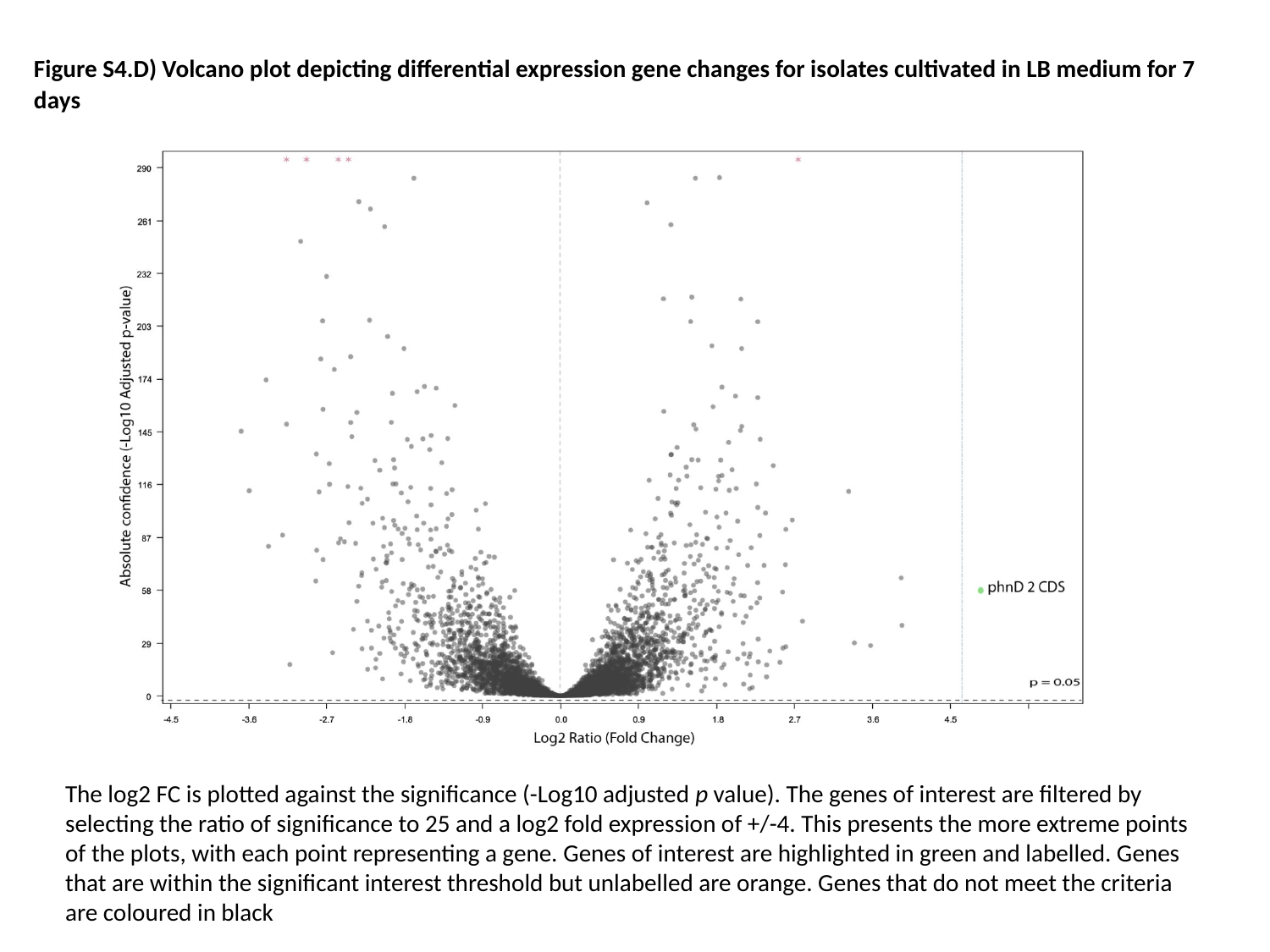

Figure S4.D) Volcano plot depicting differential expression gene changes for isolates cultivated in LB medium for 7 days
The log2 FC is plotted against the significance (-Log10 adjusted p value). The genes of interest are filtered by selecting the ratio of significance to 25 and a log2 fold expression of +/-4. This presents the more extreme points of the plots, with each point representing a gene. Genes of interest are highlighted in green and labelled. Genes that are within the significant interest threshold but unlabelled are orange. Genes that do not meet the criteria are coloured in black

## Slide 6
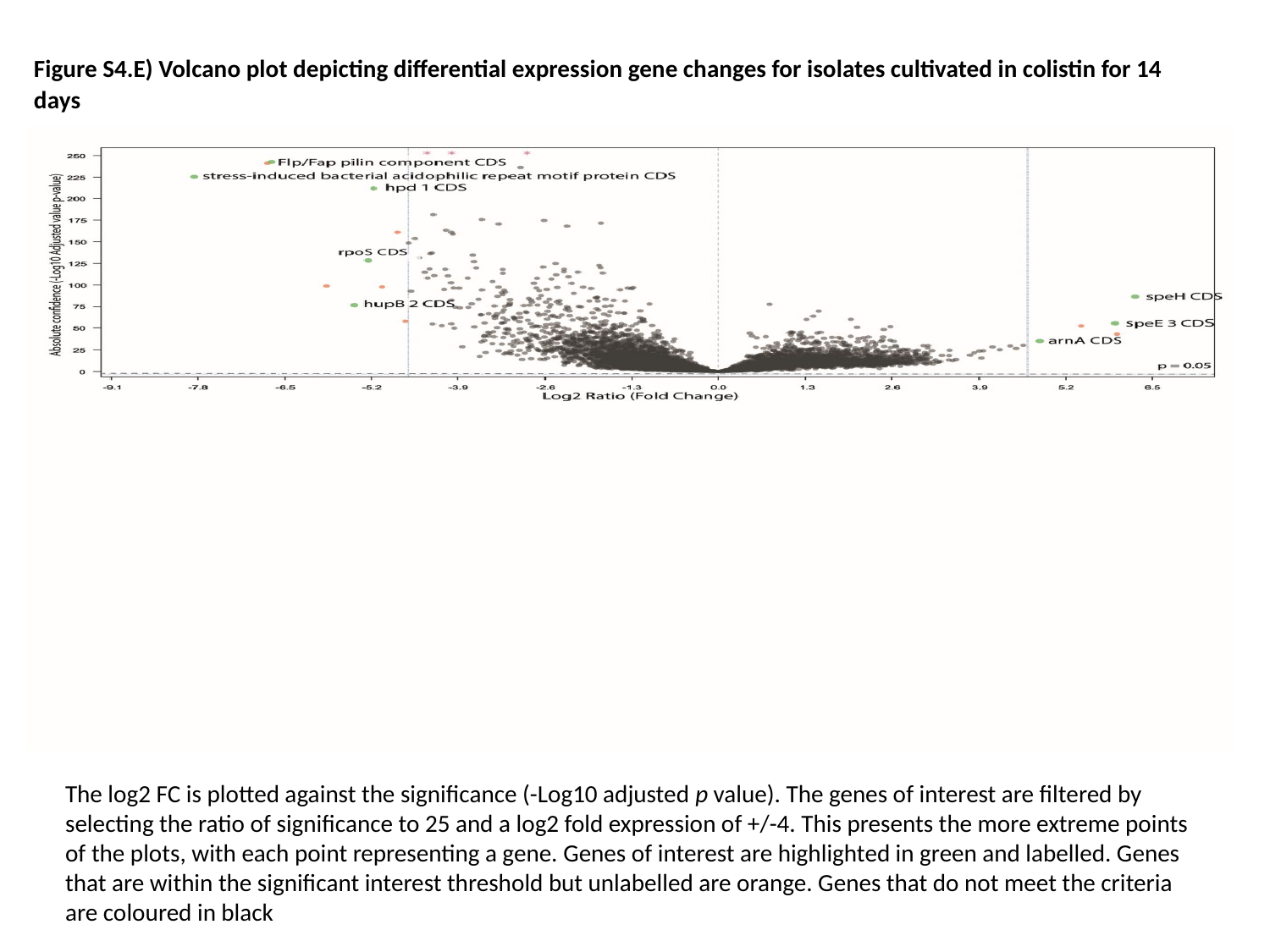

Figure S4.E) Volcano plot depicting differential expression gene changes for isolates cultivated in colistin for 14 days
The log2 FC is plotted against the significance (-Log10 adjusted p value). The genes of interest are filtered by selecting the ratio of significance to 25 and a log2 fold expression of +/-4. This presents the more extreme points of the plots, with each point representing a gene. Genes of interest are highlighted in green and labelled. Genes that are within the significant interest threshold but unlabelled are orange. Genes that do not meet the criteria are coloured in black

## Slide 7
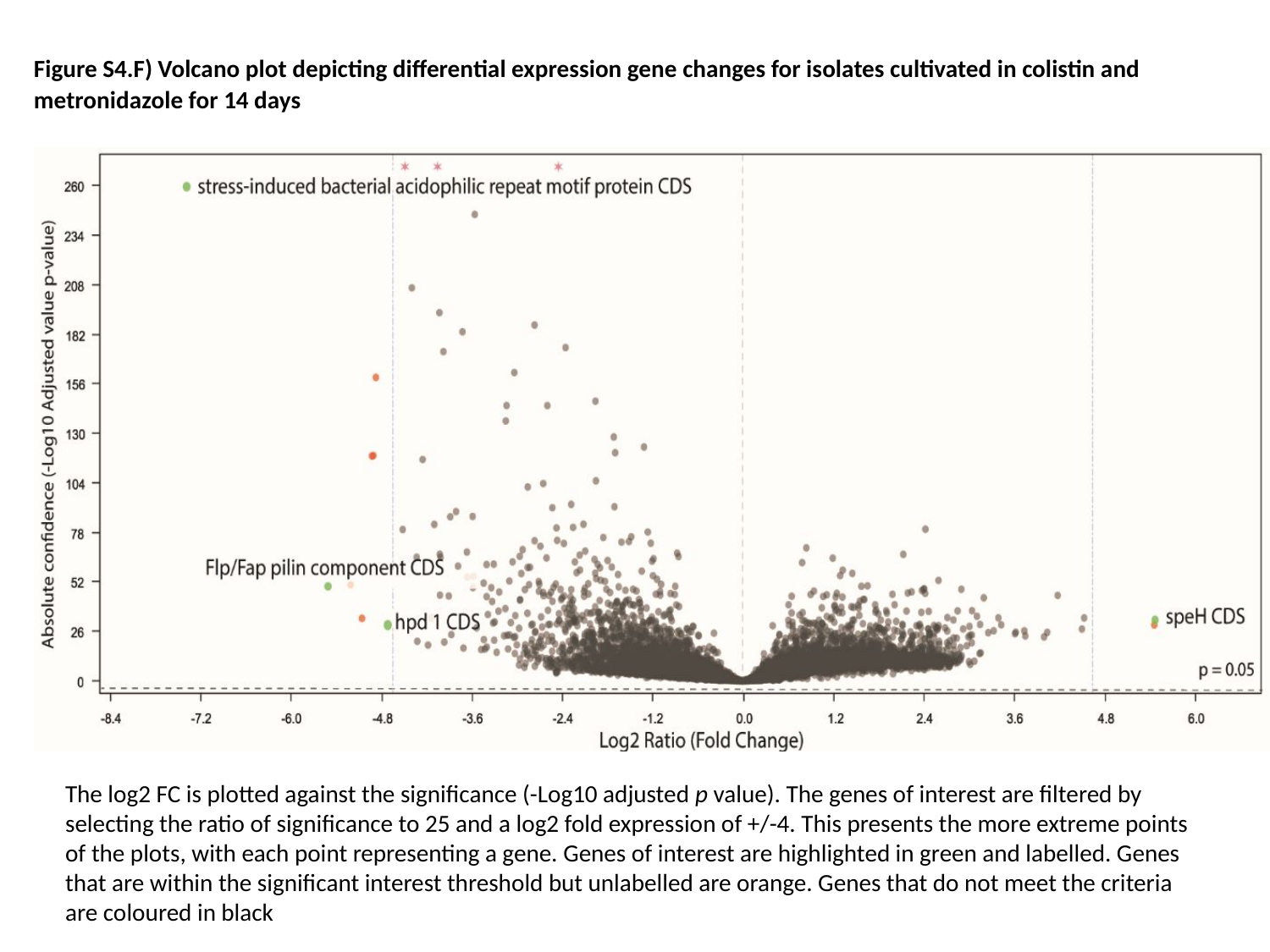

Figure S4.F) Volcano plot depicting differential expression gene changes for isolates cultivated in colistin and metronidazole for 14 days
The log2 FC is plotted against the significance (-Log10 adjusted p value). The genes of interest are filtered by selecting the ratio of significance to 25 and a log2 fold expression of +/-4. This presents the more extreme points of the plots, with each point representing a gene. Genes of interest are highlighted in green and labelled. Genes that are within the significant interest threshold but unlabelled are orange. Genes that do not meet the criteria are coloured in black

## Slide 8
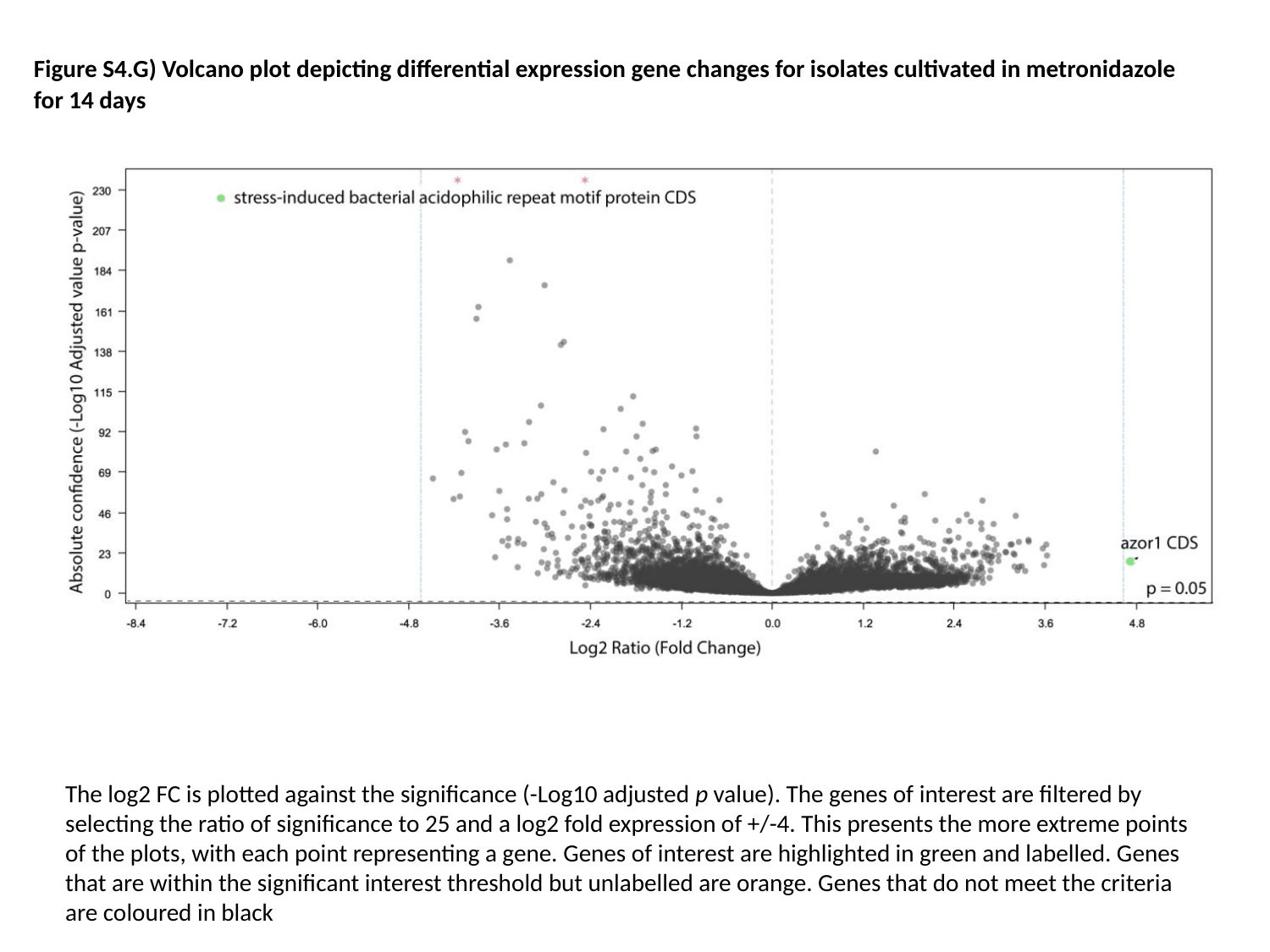

Figure S4.G) Volcano plot depicting differential expression gene changes for isolates cultivated in metronidazole for 14 days
The log2 FC is plotted against the significance (-Log10 adjusted p value). The genes of interest are filtered by selecting the ratio of significance to 25 and a log2 fold expression of +/-4. This presents the more extreme points of the plots, with each point representing a gene. Genes of interest are highlighted in green and labelled. Genes that are within the significant interest threshold but unlabelled are orange. Genes that do not meet the criteria are coloured in black

## Slide 9
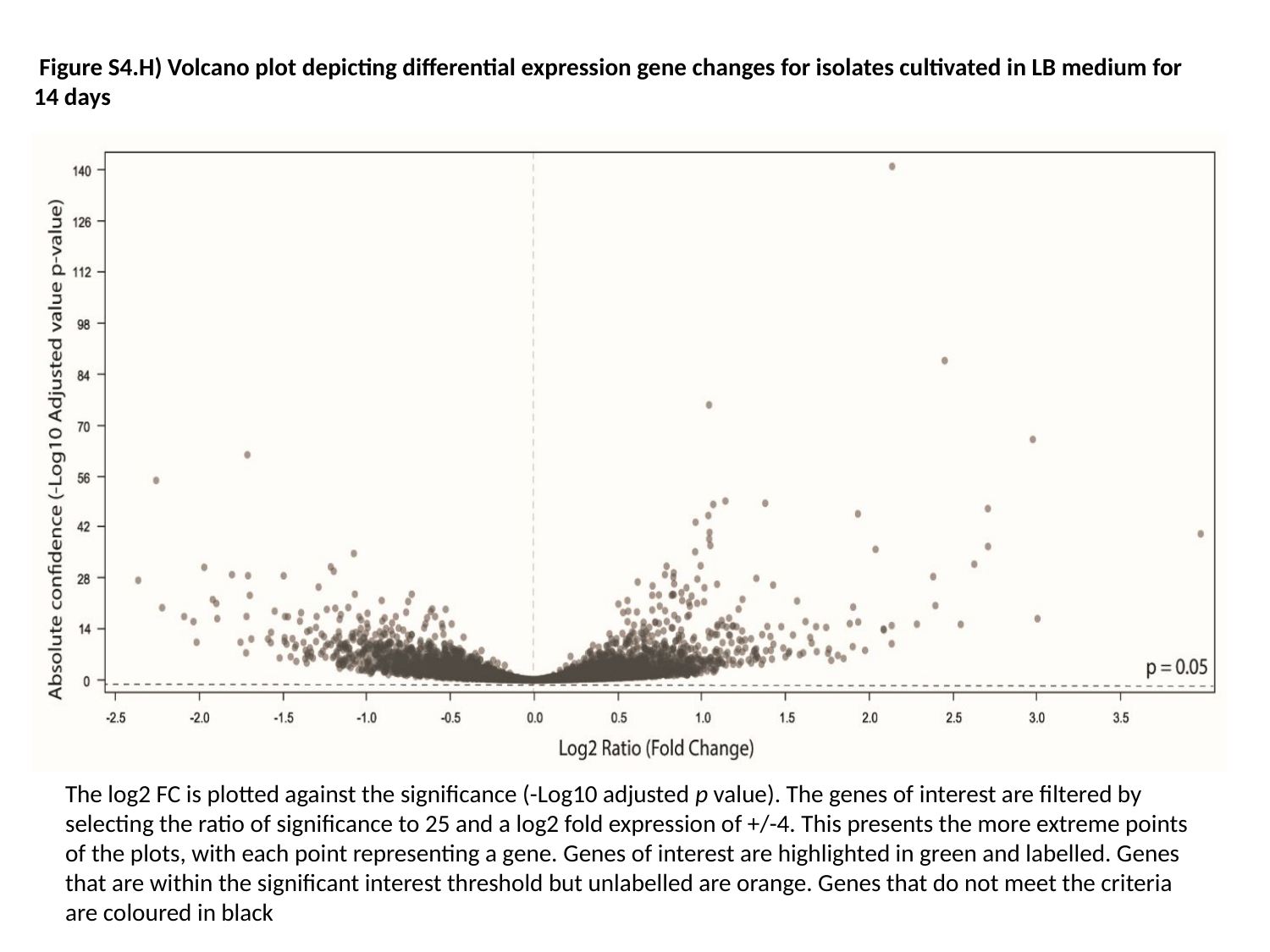

Figure S4.H) Volcano plot depicting differential expression gene changes for isolates cultivated in LB medium for 14 days
The log2 FC is plotted against the significance (-Log10 adjusted p value). The genes of interest are filtered by selecting the ratio of significance to 25 and a log2 fold expression of +/-4. This presents the more extreme points of the plots, with each point representing a gene. Genes of interest are highlighted in green and labelled. Genes that are within the significant interest threshold but unlabelled are orange. Genes that do not meet the criteria are coloured in black

## Slide 10
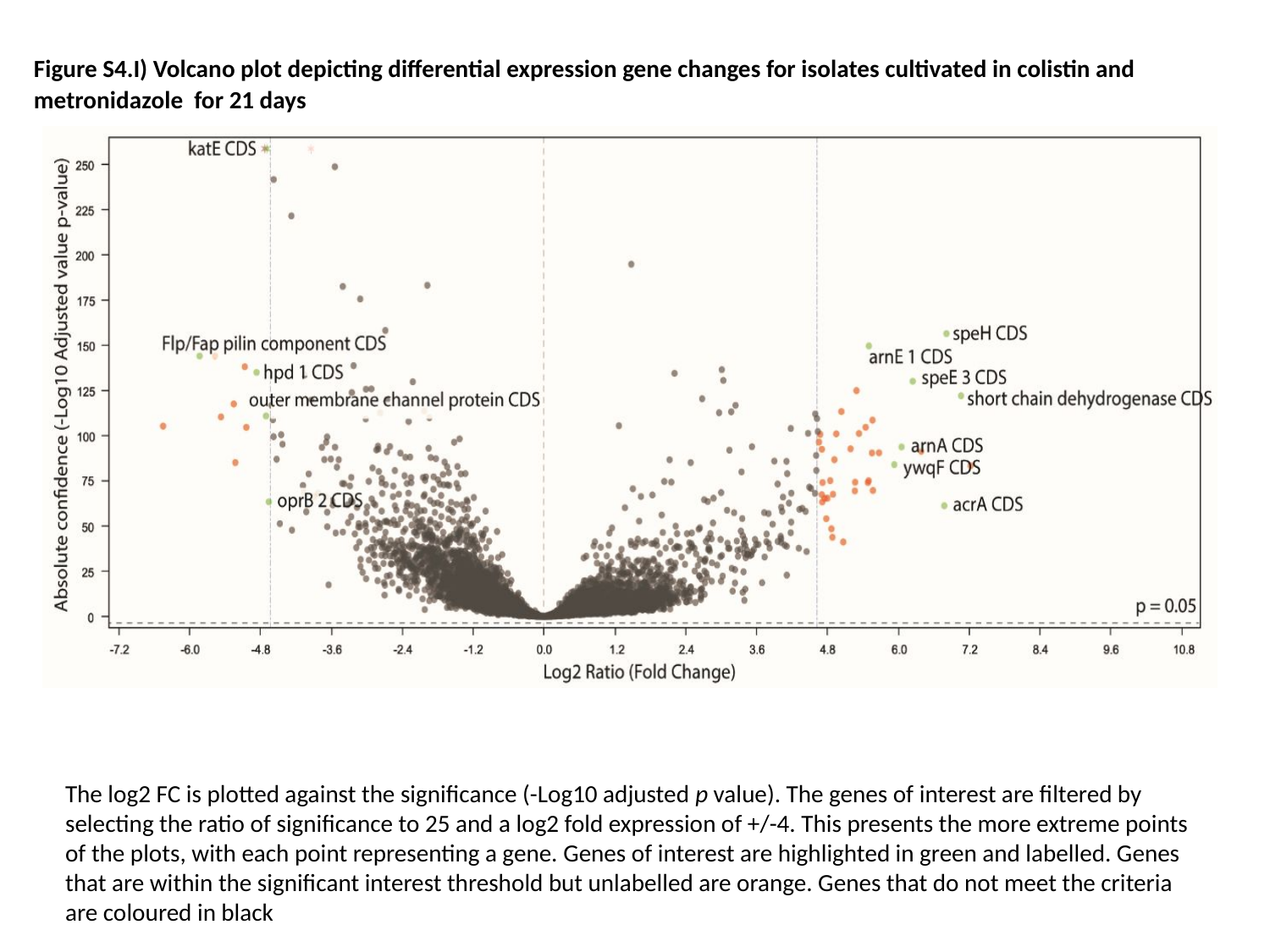

Figure S4.I) Volcano plot depicting differential expression gene changes for isolates cultivated in colistin and metronidazole for 21 days
The log2 FC is plotted against the significance (-Log10 adjusted p value). The genes of interest are filtered by selecting the ratio of significance to 25 and a log2 fold expression of +/-4. This presents the more extreme points of the plots, with each point representing a gene. Genes of interest are highlighted in green and labelled. Genes that are within the significant interest threshold but unlabelled are orange. Genes that do not meet the criteria are coloured in black

## Slide 11
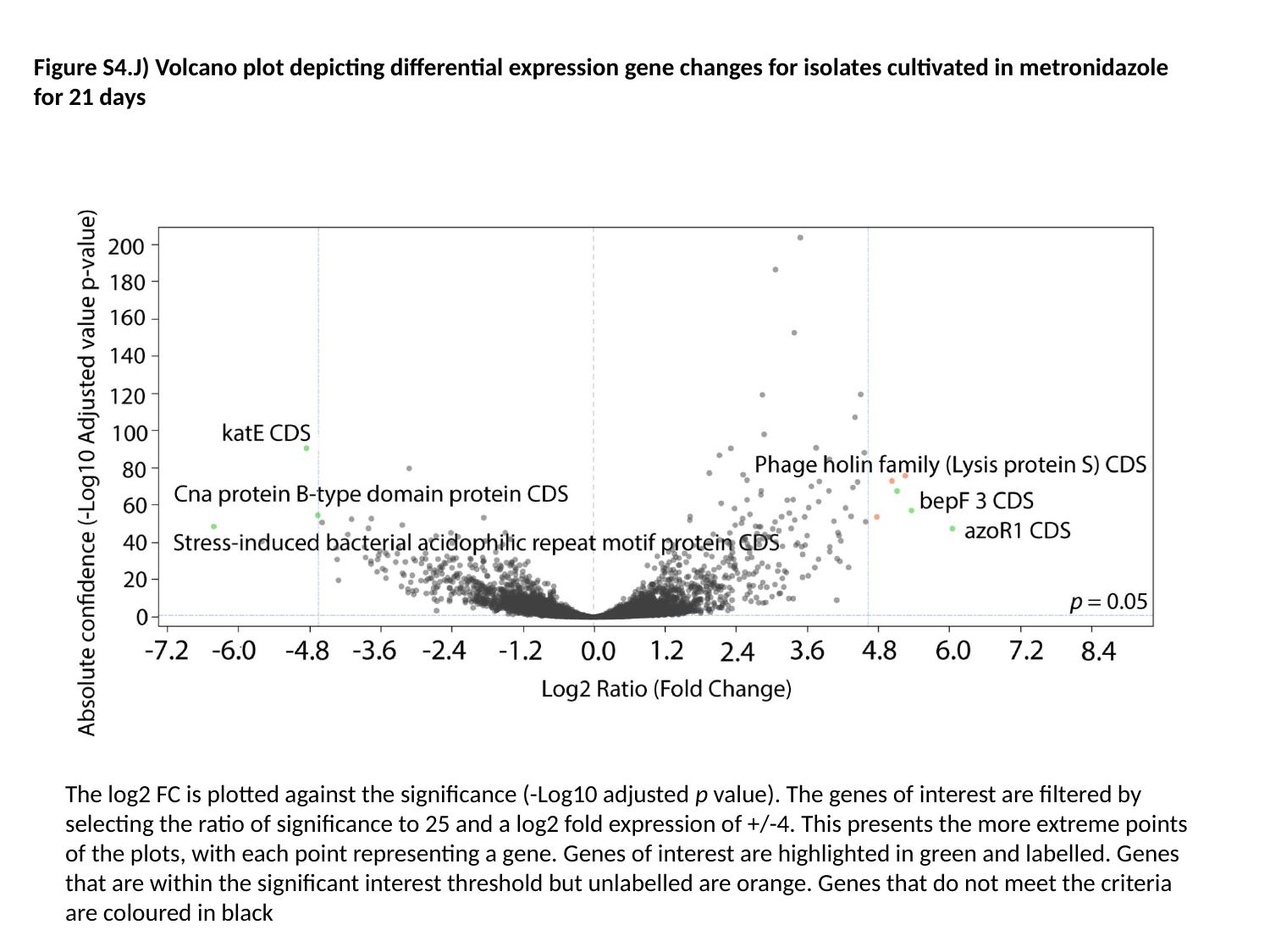

Figure S4.J) Volcano plot depicting differential expression gene changes for isolates cultivated in metronidazole for 21 days
The log2 FC is plotted against the significance (-Log10 adjusted p value). The genes of interest are filtered by selecting the ratio of significance to 25 and a log2 fold expression of +/-4. This presents the more extreme points of the plots, with each point representing a gene. Genes of interest are highlighted in green and labelled. Genes that are within the significant interest threshold but unlabelled are orange. Genes that do not meet the criteria are coloured in black
